# Longitudinal Breast MRI for Early Treatment-Response Modeling: A Comparative Study of Handcrafted Radiomics and Frozen Deep Image Embeddings for pCR Prediction

**DOI:** 10.3390/diagnostics16152349

**Published:** 2026-07-27

**Authors:** Diana Ioana Panaite, Călin Gheorghe Buzea, Florin Nedeff, Valentin Nedeff, Diana Mirilă, Mirela Panainte-Lehăduș, Claudia Tomozei, Lucian Dobreci, Constantin Volovăț, Cristian Constantin Volovăț, Roxana Irina Iancu, Maricel Agop, Lăcrămioara Ochiuz, Simona Ruxandra Volovăț

**Affiliations:** 1 Department of Medical Oncology-Radiotherapy, University of Medicine and Pharmacy “Grigore T. Popa” Iași, 700115 Iași, Romania; dianaiboboc@gmail.com (D.I.P.); volovat.constantin@umfiasi.ro (C.V.); simonavolovat@gmail.com (S.R.V.); 2 National Institute of Research and Development for Technical Physics, IFT Iași, 700050 Iași, Romania; calinb2003@yahoo.com; 3 Clinical Emergency Hospital “Prof. Dr. Nicolae Oblu” Iași, 700309 Iași, Romania; 4 Department of Environmental Engineering, Mechanical Engineering and Agritourism, Faculty of Engineering, “Vasile Alecsandri” University of Bacău, 600115 Bacău, Romania; florin_nedeff@ub.ro (F.N.); vnedeff@ub.ro (V.N.); mirelap@ub.ro (M.P.-L.); claudia.tomozei@ub.ro (C.T.); luciandd@yahoo.com (L.D.); m.agop@yahoo.com (M.A.); 5 Department of Radiology, University of Medicine and Pharmacy “Grigore T. Popa” Iași, 700115 Iași, Romania; cristian.volovat@yahoo.com; 6 Oral Pathology Department, Faculty of Dental Medicine, University of Medicine and Pharmacy “Grigore T. Popa” Iași, 700115 Iaşi, Romania; 7 “St. Spiridon” Emergency Hospital, 700111 Iaşi, Romania; 8 Faculty of Medicine, University of Medicine and Pharmacy “Grigore T. Popa” Iași, 700115 Iași, Romania; ochiuzd@yahoo.com

**Keywords:** breast MRI, neoadjuvant therapy, pathologic complete response, longitudinal imaging, radiomics, deep learning, deep image embeddings, diagnostic imaging, prognostic imaging biomarkers, treatment-response modeling, breast cancer

## Abstract

**Background/Objectives:** Early prediction of pathologic complete response (pCR) during neoadjuvant therapy could improve diagnostic assessment of treatment response, adaptive treatment monitoring, and early prognostic stratification in breast cancer, but longitudinal public MRI archives are not immediately ready for reproducible paired-image modeling. The aim of this study was to develop a reproducible longitudinal breast MRI workflow from the public ACRIN 6698/BMMR2 archive and to compare handcrafted radiomics with frozen deep image embeddings for early treatment-response modeling and imaging-based pCR prediction. **Methods:** The raw archive was reduced to a validated 183-patient cohort with matched T0 and T1 cropped DCE image–mask pairs, preserved predefined train/test assignment, and binary pCR labels. Handcrafted radiomic features were extracted at T0 and T1 using a MIRP-based workflow, longitudinal delta descriptors were constructed, and train-only cleaning, filtering, and feature selection were applied before predictive modeling. In parallel, lesion-centered paired 2.5D tensors were generated for image-based deep analysis. Exploratory end-to-end paired deep-learning models were evaluated but showed substantial overfitting. Therefore, a frozen pretrained ResNet18 feature-extraction strategy was used to derive T0, T1, DELTA, and AVG image embeddings, which were then modeled using train-only selection and classical classifiers. **Results:** In the handcrafted radiomics branch, the strongest refined model was the T1-only random forest model with 10 selected features, achieving an AUROC of 0.670 and an AUPRC of 0.405 on the independent test subset. In the frozen deep-embedding branch, the best configuration by AUROC was the DELTA-only random forest model with 30 selected features (AUROC 0.672, AUPRC 0.408), whereas the strongest configuration by AUPRC was the AVG-only logistic regression model with 100 selected features (AUROC 0.666, AUPRC 0.472). End-to-end paired deep-learning models were technically feasible but were not retained because of marked overfitting. **Conclusions:** A complex public longitudinal breast MRI archive can be converted into a reproducible modeling cohort for early response assessment. Within this framework, handcrafted radiomics and frozen deep image embeddings yielded comparable exploratory discrimination but emphasized different representation spaces: the strongest handcrafted signal was concentrated in the early-treatment T1 phenotype, whereas the strongest deep signal was concentrated in latent longitudinal change and integrated paired-image state. These findings indicate that longitudinal breast MRI-derived biomarkers warrant further investigation for early treatment-response assessment; however, the present models remain exploratory and require independent external validation before any clinical application.

## 1. Introduction

Neoadjuvant systemic therapy plays a major role in the management of biologically aggressive and locally advanced breast cancer because it can both reduce tumor burden before surgery and provide an in vivo test of treatment sensitivity. Within this setting, pathologic complete response (pCR) has become an important endpoint in translational studies and drug-development frameworks, particularly in high-risk early-stage disease. At the same time, pCR is not a universally sufficient surrogate for long-term outcome across all breast cancer subtypes and treatment contexts, which means that early imaging-based prediction of pCR is clinically relevant but methodologically nontrivial [1,2,3,4,5,6].

Breast MRI is particularly attractive for early response assessment because it can capture treatment-related changes in lesion burden, morphology, internal heterogeneity, and enhancement behavior before surgery. Serial MRI measurements have shown value in the neoadjuvant setting, and prior work from the ACRIN/I-SPY imaging program demonstrated that MRI-derived functional tumor volume and related longitudinal imaging biomarkers contain meaningful response-associated information during therapy [7,8,9]. Public resources derived from this framework, including ACRIN 6698 and the BMMR2 challenge data, provide an important opportunity for reproducible methodological work in longitudinal response modeling [10,11,12].

From a diagnostic and prognostic perspective, this problem is clinically important because early imaging-based identification of likely responders and likely non-responders could support more informative response assessment during therapy, refine risk stratification before surgery, and contribute to non-invasive clinical decision support in neoadjuvant breast cancer management. In this setting, longitudinal MRI is relevant not only as a tool for descriptive imaging follow-up, but also as a potential source of diagnostic biomarkers and early prognostic indicators of treatment effectiveness.

Radiomics offers one route for converting MRI into quantitative biomarkers by extracting predefined image descriptors that summarize lesion intensity, shape, and texture. This approach is attractive because it transforms complex lesion phenotype into analyzable numerical variables, while standardization efforts such as the Image Biomarker Standardisation Initiative have improved feature definition and software comparability across studies [13]. Recent MRI-radiomics studies and meta-analyses in the neoadjuvant setting support the potential of radiomics for pCR prediction, while also highlighting substantial variability in methodology, segmentation, and validation quality [6,14,15,16,17,18]. However, radiomics also faces well-known challenges in retrospective cohorts with limited sample size, including high dimensionality, strong feature redundancy, susceptibility to preprocessing choices, and instability when train/test separation and feature selection are not handled rigorously [13].

In parallel, machine learning and deep learning have been increasingly used for MRI-based prediction of neoadjuvant treatment response in breast cancer. Prior studies have reported encouraging results using handcrafted MRI feature sets, multiparametric MRI, and deep learning models for response-related classification tasks [5,11,19,20,21,22,23,24,25,26,27,28,29,30]. Yet deep models also introduce their own challenges: they are highly flexible, data-hungry, and prone to overfitting when trained end-to-end on modest single-cohort datasets. For this reason, frozen pretrained feature extractors may represent a more realistic alternative than fully optimized end-to-end image models in small longitudinal MRI studies [11].

A further unresolved question is whether handcrafted and deep image representations capture the same response information or instead emphasize different aspects of treatment evolution. In longitudinal imaging, predictive signal may reside in baseline phenotype, early-treatment phenotype, explicit temporal change, or integrated paired-image state. This distinction is especially relevant in datasets derived from neoadjuvant adaptive-treatment frameworks, where serial MRI was acquired precisely to detect meaningful early response patterns [7,10,11,12].

The present study was designed to address these issues in a staged and reproducible way. Using the public ACRIN 6698/BMMR2 archive, we first constructed a validated longitudinal cohort with matched T0 and T1 cropped DCE image–mask pairs and preserved predefined training/testing separation. We then developed a handcrafted radiomics benchmark based on T0, T1, and longitudinal delta descriptors, and compared it with an image-based deep representation branch built from lesion-centered paired T0/T1 2.5D tensors. Because exploratory end-to-end paired deep-learning models showed marked overfitting, we subsequently evaluated a frozen pretrained ResNet18 embedding strategy as the principal deep comparator. The aims of the study were therefore twofold: first, to establish a reproducible longitudinal preprocessing and modeling workflow for this public breast MRI archive; and second, to determine whether early treatment-response signal is expressed differently in handcrafted radiomic features and frozen deep image embeddings for pCR prediction [10,11,12,13].

## 2. Materials and Methods

### 2.1. Study Design and Public Data Source

This study was designed as a retrospective secondary analysis of a public longitudinal breast MRI dataset acquired in the neoadjuvant treatment setting. Imaging and accompanying clinical data were obtained from the ACRIN 6698/BMMR2 collection, using the BMMR2 Training set, the BMMR2 Testing set, and the Full-Collection Ancillary Patient Information spreadsheet [10,11,12]. The goal of the present work was to transform the large raw public archive into a reproducible, analysis-ready cohort suitable for longitudinal MRI-based modeling of treatment response, with pathologic complete response (pCR) as the primary endpoint.

Rather than processing the entire parent archive directly, we adopted a staged workflow in which the predefined BMMR2 train/test subsets were used as the initial working cohort. This preserved the original benchmark-style split and facilitated a reproducible pipeline for downstream image selection, segmentation matching, feature extraction, and model development.

**Figure 1:**
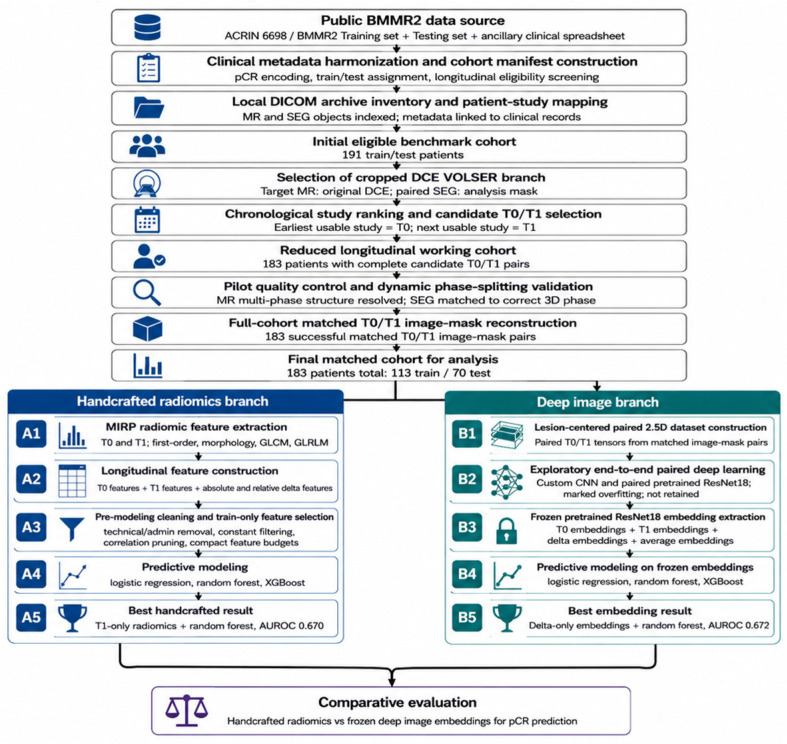
Overall study workflow for longitudinal cohort construction and comparative handcrafted/deep modeling. The public ACRIN 6698/BMMR2 archive was first harmonized at the clinical-metadata level and linked to the local DICOM inventory to define the benchmark cohort. After selection of the cropped DCE VOLSER branch, chronological study ranking, candidate T0/T1 assignment, pilot validation, dynamic phase-splitting, and full-cohort matched image–mask reconstruction, a final 183-patient matched longitudinal cohort was obtained. From this cohort, two parallel analytical branches were developed: a handcrafted radiomics branch based on MIRP feature extraction, longitudinal feature construction, train-only cleaning/selection, and predictive modeling; and a deep image branch based on lesion-centered paired 2.5D tensors, exploratory end-to-end paired deep learning, frozen pretrained ResNet18 embedding extraction, and downstream predictive modeling. The final stage consisted of comparative evaluation of handcrafted radiomics and frozen deep image embeddings for pCR prediction.

To facilitate overview of the full analytical design, the major preprocessing, radiomics, and deep image-representation steps are summarized in Figure 1.

### 2.2. Cohort Eligibility and Clinical Metadata Processing

As outlined in Figure 1, the workflow began with harmonization of the ancillary clinical metadata and construction of a machine-readable cohort manifest. Column names were standardized, clinically relevant fields were renamed into analysis-ready labels, and the original response annotation was converted into a binary pathologic complete response variable, with pCR encoded as 1 and non-pCR as 0. A separate indicator was created to document pCR-label availability. The manifest also preserved the longitudinal acquisition indicators for MRI timepoints T0–T3 and the quality-control fields available in the ancillary spreadsheet.

Initial cohort eligibility required: (i) assignment to the predefined BMMR2 training or testing subset; (ii) availability of a non-missing binary pCR label; and (iii) documentation of both T0 and T1 examinations in the clinical metadata. For inclusion in the final image-analysis cohort, patients were additionally required to have complete candidate T0 and T1 image–segmentation pairs on the selected cropped-DCE VOLSER branch and successful phase-matched image–mask reconstruction at both timepoints. Patients who did not meet these criteria were excluded from the final modeling cohort.

The ancillary clinical spreadsheet initially contained 385 patients. Among these, 191 met the initial clinical eligibility criteria, comprising 117 patients from the predefined training subset and 74 patients from the predefined testing subset. Of these 191 patients, 183 had complete candidate T0/T1 image–segmentation pairs on the selected cropped-DCE VOLSER branch. Cohort-wide phase matching, matched image–mask reconstruction, and lesion-centered 2.5D tensor generation were successful in all 183 patients. The final modeling cohort therefore consisted of 113 training patients and 70 independent testing patients.

The training subset included 78 non-pCR and 35 pCR cases, whereas the testing subset included 50 non-pCR and 20 pCR cases. Overall, the final cohort contained 128 non-pCR and 55 pCR patients. The numerical cohort flow and class distribution are summarized in Table 1.

The analysis was restricted to T0 and T1 because the primary objective was early treatment-response assessment using the first on-treatment MRI examination. Later T2 and T3 examinations occur further along the treatment pathway and may be influenced by cumulative treatment exposure, treatment adaptation, and reduced longitudinal availability. Their inclusion would therefore address a different prediction setting and could reduce the number of patients with complete matched examinations. T2- and T3-based temporal modeling was considered beyond the scope of the present early-response study and remains an important direction for future work.

To examine potential imaging-availability selection bias, the available clinical characteristics of the 183 included patients were compared with those of the eight patients excluded because of incomplete candidate T0/T1 imaging pairs. Age and maximum lesion diameter were compared using the Mann–Whitney U test, whereas pCR distribution was compared using Fisher’s exact test. Dataset split, race, lesion type, hormone-receptor/HER2 category, and SBR grade were summarized descriptively. Formal statistical testing of the remaining categorical characteristics was not emphasized because the excluded group contained only eight patients.

### 2.3. Local DICOM Archive Inventory

Because the raw imaging archives were very large, all initial archive inspection was performed locally rather than in a cloud notebook environment. A DICOM inventory procedure was implemented to recursively scan the downloaded BMMR2 training and testing directories and extract metadata from each DICOM object. For each file, the inventory recorded the patient identifier, modality, study UID, series UID, SOP Instance UID, study date, study time, series description, protocol name, scanner vendor/model information, and absolute file path.

The resulting inventory confirmed the presence of the complete 191-patient train/test imaging cohort, comprising approximately 1.48 million MR files and 1191 SEG objects. This step was critical because it established that the local downloads contained both the expected MRI data and the corresponding segmentation objects required for ROI-based image analysis.

The DICOM inventory was implemented in Python. The local preprocessing workflow used pydicom for DICOM header parsing and SEG pixel-array decoding, pandas for tabular data management, NumPy for array operations, SimpleITK for MR-volume reconstruction, image input/output, resizing, and NIfTI conversion [31], and SciPy for connected-component analysis. Radiomic extraction was performed using MIRP, classical machine-learning analyses were implemented using scikit-learn and XGBoost, and the exploratory deep-learning analyses used PyTorch and torchvision. The saved execution environment documented Python 3.12.13 and SimpleITK version 2.5.5. The software environment comprised Python 3.12, pandas version 2.2.2, NumPy version 2.0.2, SimpleITK version 2.5.5, SciPy version 1.16.3, scikit-learn version 1.6.1, XGBoost version 3.3.0, PyTorch version 2.11.0, and torchvision version 0.26.0.

The inventory procedure followed the following steps: (1) recursively enumerate all files within the predefined training and testing directories; (2) identify DICOM objects using the standard file preamble; (3) read each DICOM header without loading pixel data; (4) extract patient, study, series, SOP-instance, modality, acquisition-time, protocol, manufacturer, image-dimension, and file-path information; (5) store one record per DICOM object in a file-level inventory; and (6) aggregate the file-level records into series-level and patient-level summary tables used for subsequent image–segmentation matching. Files that could not be read were recorded without interrupting the complete archive scan.

### 2.4. Patient-Level Mapping Between Clinical Metadata and Imaging Archive

To connect the ancillary spreadsheet with the raw imaging archive, the corrected cohort manifest was merged with the DICOM inventory using patient identifiers and train/test split membership. From the file-level inventory, two additional derived tables were generated: a series-level summary, aggregating files into unique MR or SEG series, and a patient-level summary, describing the number of studies, number of series, and presence of MR and SEG objects per patient.

Patient eligibility was defined according to the criteria reported in Section 2.2. At the patient-mapping stage, all 191 initially eligible patients had both MR and SEG objects identified in the local archive.

### 2.5. Selection of the Initial Imaging Branch

Inspection of the series-level metadata revealed that the archive contained multiple imaging branches, including raw dynamic contrast-enhanced MRI, cropped DCE-derived VOLSER images, diffusion-weighted imaging, ADC-derived series, and multiple segmentation families. For the first radiomics pipeline, we deliberately selected a practical and internally coherent imaging branch instead of attempting to use all available modalities simultaneously.

The preferred initial branch was defined by the recurring MR series description:

- “ISPY2: VOLSER: uni-lateral cropped: original DCE”

and the corresponding segmentation description:

- “ISPY2: VOLSER: uni-lateral cropped: Analysis Mask”.

This branch was chosen because it provided already cropped uni-lateral DCE images together with paired analysis masks, thereby reducing preprocessing complexity and minimizing the burden of full-volume handling. The purpose of this first-stage choice was to establish a clean, reproducible pipeline before considering expansion to other modalities such as DWI or ADC. DWI and ADC were not excluded because they lack potential predictive relevance. They were omitted to maintain a single internally coherent longitudinal representation for the controlled comparison between handcrafted radiomics and deep image embeddings. Their inclusion would require a separate modality-specific workflow addressing sequence availability, geometric correspondence, registration to the selected DCE phase, segmentation transfer, intensity harmonization, and modality-specific feature extraction. Multimodal integration was therefore considered a distinct extension rather than a minor addition to the present study.

### 2.6. Chronological Ranking of Studies and Assignment of Candidate T0 and T1 Pairs

For each patient, studies were grouped at the study level and ranked chronologically using study date and study time. Within each study, we detected whether both a target cropped DCE MR series and a corresponding analysis-mask segmentation were present. Studies containing both elements were marked as usable candidate studies.

For each patient, the earliest usable study was provisionally assigned as candidate T0, and the next usable study as candidate T1. This produced both a study-level candidate table and a patient-level table listing the selected T0 and T1 MR and SEG series UIDs.

Using this approach, 183 of the 191 BMMR2 train/test patients were found to have complete candidate T0 and T1 pairs on the preferred cropped-DCE branch. The remaining 8 patients lacked a complete T0/T1 pair on this initial route and were excluded from the first-stage extraction workflow. Accordingly, the reduced working cohort for subsequent image-level processing consisted of 183 patients.

All eight excluded patients lacked an identifiable candidate T0 image–segmentation pair and an identifiable candidate T1 image–segmentation pair on the selected cropped-DCE VOLSER branch. The excluded group comprised four patients from the predefined training subset and four from the testing subset and included four pCR and four non-pCR cases. Median age was 44.50 years [interquartile range, 35.75–57.25] in the excluded group and 48.00 years [41.50–56.00] in the included cohort (*p* = 0.601). Median maximum lesion diameter was 3.70 [3.23–5.05] and 3.50 [2.80–5.05], respectively (*p* = 0.736). The pCR distribution did not differ significantly between the excluded and included groups (*p* = 0.255, Fisher’s exact test). The remaining available clinical characteristics are summarized descriptively in Table S2. Because only eight patients were excluded, these comparisons had limited statistical power and were not interpreted as evidence that selection bias was absent.

### 2.7. File-Level Mapping of Selected Series

After patient-level selection of candidate T0 and T1 studies, the selected MR and SEG series UIDs were mapped back to the full DICOM inventory to recover the exact file paths belonging to each chosen series. This produced a file-level mapping table containing all DICOM files corresponding to:

- T0 MR
- T0 SEG
- T1 MR
- T1 SEG.

A patient-level quality-control summary was then derived from this file map to verify the presence of all four required series for each selected patient. This step confirmed that all 183 selected patients had complete T0_MR, T0_SEG, T1_MR, and T1_SEG series present, thereby validating the integrity of the reduced working cohort at the file level.

### 2.8. Pilot Validation of the Image–Segmentation Preprocessing Workflow

Before cohort-wide processing, the image–segmentation workflow was validated using a pilot subset of five patients that included both pCR and non-pCR cases. For each patient, the selected T0 and T1 cropped-DCE MR series and corresponding SEG objects were retrieved and assessed for readability, dimensional compatibility, and lesion-mask content. All selected MR and SEG objects were successfully loaded, and the segmentation objects contained valid, non-empty binary data.

Pilot reconstruction showed that the selected cropped-DCE series contained multiple dynamic three-dimensional phases, whereas each SEG object corresponded to a single phase. Candidate phases were therefore identified using temporal metadata, when available, and repeated slice-position patterns. The phase whose through-plane dimension matched that of the corresponding segmentation was selected independently at T0 and T1. A matching phase was successfully identified for all five pilot patients at both timepoints.

The SEG objects were decoded into binary masks, and both the original mask and a cleaned mask retaining the largest connected component were generated. Image–mask dimensional compatibility, connected-component structure, lesion bounding-box extent, and the fraction of segmented voxels contained in the largest component were examined. The largest component contained essentially all segmented voxels in the pilot cases, confirming that the masks represented coherent lesion regions. Apparent rectangular structures observed during direct DICOM visualization were therefore interpreted as display-related artifacts rather than defects in the binary regions used for analysis. The validated procedure was subsequently applied to the complete cohort.

### 2.9. Cohort-Wide Matched Image–Mask Generation and Quality Control

Following pilot validation, the phase-splitting and image–segmentation matching workflow was applied to the complete 183-patient cohort at both T0 and T1. For each selected cropped-DCE MR series, temporal structure was identified using explicit temporal metadata, when available, or repeated z-position patterns. Each series was then separated into candidate three-dimensional dynamic phases.

The corresponding SEG object was decoded into a binary segmentation volume, and its number of slices was compared with that of each candidate DCE phase. The phase whose through-plane dimension matched the segmentation was selected independently at T0 and T1. The selected MR phase was saved as a NIfTI volume, while the SEG object was preserved as a raw binary mask. A cleaned mask was additionally generated by retaining the largest connected component.

Cohort-wide quality control included confirmation of successful phase identification, image–mask dimensional compatibility, connected-component structure, and the fraction of segmented voxels contained in the largest component. A valid matching phase and a matched image–mask pair were obtained at both T0 and T1 for all 183 patients. The largest connected component contained virtually all segmented voxels in nearly all cases, indicating that the lesion masks were spatially coherent and minimally affected by disconnected artifacts. Cleaned masks were therefore retained for radiomic analysis, while the raw masks were preserved for traceability.

The final longitudinal imaging cohort comprised 183 patients, including 113 training patients and 70 independent testing patients. For each patient, the finalized dataset contained a matched cropped-DCE image volume and lesion mask at both T0 and T1, together with the predefined BMMR2 split assignment, binary pCR endpoint, baseline clinical variables, and patient-level quality-control metadata. These data constituted the final imaging substrate for radiomic feature extraction, longitudinal feature construction, and deep image-representation analysis.

### 2.10. MIRP Radiomic Feature Extraction

Following construction of the matched longitudinal image–mask dataset, radiomic feature extraction was performed from the phase-matched cropped DCE image volumes using the corresponding cleaned binary lesion masks. Feature extraction was carried out separately for T0 and T1 while preserving the predefined BMMR2 training and testing assignment and the binary pCR endpoint.

A standardized MIRP-based extraction workflow was used [13,32]. Intensity discretization was performed using a fixed-bin-number approach with 32 bins. To maintain a controlled first-pass feature space while limiting unnecessary computational burden, extraction was restricted to four feature families: first-order statistical features, morphology features, grey-level co-occurrence matrix features, and grey-level run-length matrix features.

Radiomic extraction was first executed independently within the predefined testing and training subsets, producing four primary outputs: T0-testing, T1-testing, T0-training, and T1-training. Extraction was successful in both subsets. Radiomic features were obtained for 70 testing patients and 113 training patients at both T0 and T1, yielding complete radiomic coverage for the full validated 183-patient longitudinal cohort.

### 2.11. Construction of Full-Cohort T0, T1, and Longitudinal Delta Feature Tables

After split-wise extraction, the T0-training and T0-testing tables were concatenated to produce a unified full-cohort T0 radiomic table, and the T1-training and T1-testing tables were concatenated analogously to produce a unified full-cohort T1 radiomic table. Duplicate patient entries were removed if present, and T0 and T1 tables were aligned using patient identifiers. This alignment confirmed complete patient-level concordance between timepoints across the full cohort of 183 patients.

For each radiomic feature available at both timepoints, longitudinal delta descriptors were then computed. Two complementary delta representations were generated: (i) an absolute delta, defined as T1 minus T0, and (ii) a relative delta, defined as the T1–T0 difference normalized by the absolute T0 value whenever the denominator was non-zero. This procedure yielded a longitudinal feature space explicitly representing early treatment-associated imaging change.

A final merged dataset was then assembled containing: (i) preserved patient-level metadata and baseline clinical variables, (ii) T0 radiomic features, (iii) T1 radiomic features, and (iv) derived longitudinal delta features. The resulting dataset comprised 183 patients, preserving the predefined split structure of 113 training patients and 70 testing patients.

Before downstream model development, technical metadata columns related to file names, directory paths, image-series identifiers, reconstruction settings, and other non-biological processing descriptors were marked for exclusion from the analytical feature matrix. Constant or otherwise non-informative variables were likewise designated for removal during preprocessing. Thus, the merged radiomic table generated at this stage constitutes the raw quantitative substrate for the next step of the study, namely feature cleaning and construction of the final machine-learning-ready modeling matrix.

### 2.12. Pre-Modeling Feature Cleaning and Variable Screening

Before classifier development, the merged radiomic dataset underwent a dedicated pre-modeling cleaning stage designed to convert the raw quantitative table into a machine-learning-ready analytical matrix. This step was necessary because the initial merged table contained, in addition to biologically meaningful radiomic descriptors, multiple technical and administrative columns generated during image conversion and feature extraction, including file names, directory paths, image-series identifiers, processing descriptors, and acquisition-related metadata. Such variables were not considered appropriate predictive inputs because they reflected data management or reconstruction bookkeeping rather than intrinsic tumor phenotype or treatment-related imaging change.

The first part of the cleaning procedure therefore consisted of excluding non-biological technical descriptors from the candidate predictor matrix while preserving the patient identifier, predefined train/test assignment, binary pCR endpoint, and selected baseline clinical covariates. After this exclusion step, the remaining feature space was screened for variables with no analytical utility. All-NA variables were removed, and features with more than 30% missing values in the training subset were excluded. Constant features were removed, while a feature was defined as near-constant when a single value accounted for at least 95% of the training observations. Highly correlated features were subsequently pruned using an absolute pairwise correlation threshold of 0.95, calculated exclusively within the training subset. The remaining variables were ranked using training-only mutual-information relevance scores [33] before evaluation of compact feature budgets. In addition, acquisition-geometry descriptors such as voxel-size variables were excluded from the modeling matrix in order to reduce the influence of non-biological imaging protocol characteristics on predictive learning.

Because the merged dataset contained baseline T0 radiomic features, early-treatment T1 radiomic features, and derived longitudinal delta descriptors, this cleaning stage was applied across all three feature blocks in a harmonized manner. The purpose was not to alter the underlying biological information, but to reduce noise, redundancy, and instability before predictive modeling. The output of this step was the final machine-learning-ready matrix used for pCR prediction, model comparison, and explainable AI analysis.

### 2.13. Predictive Modeling Strategy

Following pre-modeling feature cleaning, the final machine-learning-ready matrix was used for supervised prediction of pathologic complete response (pCR). The predefined BMMR2 split structure was preserved throughout this stage, such that the 113-patient training subset was used for model development and the 70-patient testing subset was reserved for independent performance evaluation. This design was maintained in order to preserve the benchmark-style separation already embedded in the source dataset and to minimize information leakage between model development and final assessment.

Model development was performed in a staged comparative manner. Separate predictive representations were defined for: (i) baseline clinical variables alone; (ii) baseline T0 radiomic features alone; (iii) early-treatment T1 radiomic features alone; (iv) combined T0 and T1 radiomic features; (v) longitudinal delta radiomic features; and (vi) integrated models combining clinical covariates with baseline, early-treatment, and longitudinal imaging features. This structure was chosen to determine whether early-treatment imaging change provides incremental predictive value beyond baseline morphology and baseline clinical information.

Within the training subset, missing values were handled using training-derived imputation only, and any additional scaling or transformation steps required by a given classifier were fitted exclusively on the training data and then applied unchanged to the testing data. This approach ensured that test-set information did not influence feature preprocessing or model fitting. Model comparison was performed using a set of complementary algorithms representing both linear and non-linear learning strategies, including logistic regression, random forest, and gradient-boosted tree methods [33,34].

During the first-pass and feature-budget screening analyses, fixed classifier configurations were used to limit model-selection complexity. Logistic regression was implemented after median imputation and standard scaling, using the liblinear solver, a maximum of 5000 iterations, inverse-frequency balanced class weights, and a random seed of 42. Random forest models used median imputation, 500 trees, unrestricted maximum depth, a minimum of two observations per terminal leaf, balanced class weights, and a random seed of 42. The XGBoost gradient-boosted tree models used median imputation, 400 estimators, a maximum tree depth of 4, a learning rate of 0.03, subsampling of 0.85, column subsampling of 0.85, L2 regularization of 1.0, a binary logistic objective, and log-loss evaluation.

Feature-selected screening was performed after train-only removal of unusable and highly correlated variables and mutual-information ranking. Candidate feature budgets of 5, 10, 20, and 30 variables were evaluated in the general feature-screening stage. A subsequent targeted tuning stage was restricted to the strongest T1-only, combined T0 + T1, and clinical + T0 + T1 feature blocks. Candidate feature budgets were 5, 8, 10, 12, and 15 for the T1-only block and 10, 15, 20, and 25 for the combined blocks.

The candidate baseline clinical metadata comprised age, race, lesion type, hormone-receptor/HER2 category, SBR grade, and maximum lesion diameter. For predictive modeling, the clinical-only block comprised age and maximum lesion diameter (MRLD), which were the two continuous baseline variables that remained directly analyzable after numeric harmonization and train-side usability screening. Race, lesion type, hormone-receptor/HER2 category, and SBR grade were retained for cohort description but were not entered into the current predictive models because categorical encoding and subtype-stratified modeling were not implemented in this first-stage analysis. The clinical-only model was therefore intended as a compact non-imaging reference benchmark rather than as a comprehensive clinical prediction model.

Targeted hyperparameter optimization was performed using five-fold stratified cross-validation with shuffling and a random seed of 42, exclusively within the training subset. Grid-search selection was based on mean cross-validated AUROC. For logistic regression, the regularization parameter C was evaluated at 0.01, 0.1, 1.0, 5.0, and 10.0. For random forest, the evaluated settings comprised 300 or 600 trees; maximum depths of unrestricted, 4, 6, or 8; minimum terminal-leaf sizes of 1, 2, or 4; and maximum-feature settings of square-root or 0.5 of the available predictors. For XGBoost, the grid included 200 or 400 estimators; maximum depths of 3, 4, or 5; learning rates of 0.03, 0.05, or 0.10; subsampling fractions of 0.8 or 1.0; and column-subsampling fractions of 0.8 or 1.0. All preprocessing, feature selection, and hyperparameter selection steps were performed without access to the independent test labels. Logistic regression, random forest, and gradient boosting were selected to provide complementary regularized linear, bagged non-linear, and boosted non-linear learning strategies while limiting extensive algorithm screening in relation to the modest 113-patient training cohort.

Class imbalance was addressed in the logistic regression and random forest models using inverse-frequency balanced class weights. Synthetic minority oversampling, including SMOTE, was not applied because interpolation within the high-dimensional radiomic feature space, using only 113 development cases, could generate unstable synthetic observations and introduce an additional source of overfitting. Class weighting was therefore preferred because it preserved the observed training-data distribution while increasing the contribution of the minority pCR class during model fitting.

The primary operating-point metrics were calculated at a prespecified probability threshold of 0.5. In a secondary sensitivity analysis performed for the strongest radiomic configurations, alternative probability thresholds were selected exclusively from out-of-fold predictions generated within the training subset using five-fold stratified cross-validation. Candidate thresholds between 0.20 and 0.80 were evaluated in increments of 0.02, and the threshold maximizing the Youden index was retained. The independent test labels were not used for threshold selection.

Predictive performance was evaluated primarily on the independent testing subset. The principal discrimination metrics included the area under the receiver operating characteristic curve (AUROC) and the area under the precision–recall curve (AUPRC). Additional operating-characteristic measures included accuracy, sensitivity, specificity, precision, F1 score, and related confusion-matrix-derived quantities. Binary operating-point metrics were calculated with explicit zero-event handling, and the corresponding true-negative, false-positive, false-negative, and true-positive counts were retained for each evaluated model. Consequently, a reported precision, sensitivity, or F1 value of zero indicates that no true-positive classifications occurred at the selected probability threshold rather than a division-by-zero error or failed metric calculation. To support interpretability, feature-importance analysis and explainable AI procedures were reserved for the subsequent refinement stage, with particular interest in identifying whether longitudinal delta radiomic variables contribute materially to pCR prediction beyond baseline imaging features alone.

This modeling stage was designed as the handcrafted-feature benchmark arm of the study and was used to establish a radiomics-based reference against which subsequent image-derived deep representation strategies could be compared.

### 2.14. Construction of a Paired T0/T1 2.5D Deep Learning Dataset

To enable image-based deep representation learning in parallel with the handcrafted radiomics analyses, an additional lesion-centered paired image dataset was constructed from the final matched longitudinal cohort. This procedure used the same 183 patients described above, preserving the predefined BMMR2 training/testing split and the binary pCR endpoint, but transformed the phase-matched T0 and T1 image–mask pairs into a compact, standardized tensor representation suitable for convolutional neural network input.

For each patient and each timepoint, the cleaned binary lesion mask was used to determine the lesion bounding box and the corresponding lesion-centered spatial region within the matched cropped-DCE image volume. The axial slice range occupied by the lesion was identified from the mask, and the central lesion slice was defined as the rounded midpoint of the lesion-containing z-extent. A fixed seven-slice 2.5D representation was then constructed using the central slice, three adjacent slices above it, and three adjacent slices below it. When the requested range extended beyond the available image volume, the slice indices were clipped to the valid z-range.

An in-plane crop was defined from the lesion-mask bounding box and expanded by a fixed margin of 12 pixels in both spatial directions. Crop boundaries were clipped to the available image dimensions. The resulting cropped image volume was intensity-normalized by clipping voxel intensities to the first and 99th percentiles, followed by z-score standardization. When the standard deviation of the clipped volume was lower than 1 × 10^−8^, mean centering was applied without division by the standard deviation. Each of the seven selected axial slices was subsequently resized to 224 × 224 pixels using linear interpolation implemented in SimpleITK, and the resized slices were stacked to generate a fixed-size 2.5D tensor.

This procedure was applied independently to T0 and T1, yielding one lesion-centered T0 tensor and one lesion-centered T1 tensor for each patient. The final tensor shape at each timepoint was 7 × 224 × 224. All generated arrays were saved as patient-level files together with a dedicated manifest documenting the patient identifier, split membership, pCR label, array paths, and tensor dimensions. Quality-control logs documented processing success, crop boundaries and dimensions, lesion-center position, selected slice indices, and lesion-mask voxel content. Paired T0 and T1 2.5D tensors were generated successfully for all 183 patients, with no processing failures.

The purpose of this dataset-construction step was to create a standardized image-based representation that preserved early-treatment longitudinal information while remaining computationally tractable for small-sample deep-learning experiments. Unlike the handcrafted radiomics branch, which summarized lesion phenotype using predefined engineered descriptors, the paired 2.5D dataset supported the extraction and learning of image-derived latent representations directly from lesion-centered T0 and T1 appearance patterns.

A seven-slice representation was selected to retain broader lesion-centered through-plane context than a conventional three-slice input while preserving compatibility with two-dimensional pretrained convolutional backbones. A fully three-dimensional CNN was not adopted because the development cohort contained only 113 patients, whereas three-dimensional architectures would substantially increase the number of trainable parameters, computational requirements, and susceptibility to overfitting. Furthermore, a fully three-dimensional approach would require additional harmonization of through-plane resolution and slice geometry across examinations. The 2.5D design was therefore adopted as a pragmatic compromise between spatial context, computational tractability, reproducibility, and small-sample stability.

### 2.15. Exploratory End-to-End Paired T0/T1 Deep Learning Modeling

To examine whether treatment-response information could be learned directly from the paired longitudinal image data rather than from predefined handcrafted descriptors, an exploratory end-to-end deep learning branch was implemented using the 2.5D T0 and T1 tensor pairs described above. The purpose of this branch was not to replace the radiomics benchmark a priori, but to test whether a direct image-based representation of early treatment evolution could provide additional predictive value for pathologic complete response (pCR).

A paired-input architecture was adopted in which the T0 and T1 lesion-centered tensors were processed jointly. In this formulation, each patient contributed two matched image inputs corresponding to the pretreatment and early-treatment timepoints, respectively. The model was designed to learn latent representations of the lesion appearance at T0 and T1 and to combine these representations into a unified prediction of pCR status. This paired-input design was chosen in order to preserve the temporal ordering of the two acquisitions and to allow the model, in principle, to capture not only absolute lesion phenotype at each timepoint but also treatment-associated change reflected in the relation between T0 and T1.

Model development was restricted to the predefined training subset of 113 patients. Within this training subset, an additional internal validation split was created using stratified sampling in order to monitor optimization behavior and reduce the risk of selecting models solely on the basis of training performance. The independent 70-patient testing subset remained fully held out and was reserved for final performance assessment only. This separation was preserved throughout all exploratory deep learning experiments to minimize leakage between model development and final evaluation.

Two exploratory end-to-end paired-image architectures were evaluated. The first was a compact custom shared-weight convolutional network. Each seven-channel T0 or T1 tensor was processed by the same encoder comprising five consecutive 3 × 3 convolutional blocks with 16, 32, 64, 96, and 128 output channels, respectively. Each block included batch normalization and ReLU activation; max pooling followed the first four blocks, and adaptive global average pooling generated a 128-dimensional representation. The T0 representation, T1 representation, and their element-wise difference were concatenated into a 384-dimensional vector and processed by a fully connected 384–128–32–1 prediction head with dropout rates of 0.30 and 0.20. Training used class-weighted binary cross-entropy, the Adam optimizer, a learning rate of 1 × 10^−3^, weight decay of 1 × 10^−4^, a batch size of 16, a maximum of 60 epochs, and early stopping with a patience of 10 epochs.

The second exploratory architecture used an ImageNet-pretrained ResNet18 backbone with shared weights for the T0 and T1 inputs [35,36,37]. The original three-channel input layer was adapted to accept the seven-slice 2.5D tensors. The pretrained convolutional weights were averaged across the original RGB input-channel dimension and the resulting mean filters were repeated across the seven input channels. The earlier ResNet18 blocks were frozen, whereas the complete Layer 4 block and the paired fusion head remained trainable. The 512-dimensional T0 and T1 representations and their element-wise difference were concatenated before binary classification. The partially fine-tuned model was optimized using AdamW with a learning rate of 3 × 10^−4^, weight decay of 1 × 10^−4^, and a batch size of 16 for a maximum of 40 epochs. A ReduceLROnPlateau scheduler reduced the learning rate by a factor of 0.5 after three epochs without validation-AUROC improvement, and early stopping was applied with a patience of eight epochs.

Both architectures were trained using class-weighted binary cross-entropy, with the positive-class weight calculated exclusively from the class distribution of the training subset. The compact custom CNN was optimized using Adam, whereas the partially fine-tuned ResNet18 model was optimized using AdamW with weight decay and a learning-rate scheduler. Model development was monitored on the internal stratified validation subset, and the model state associated with the highest validation AUROC was retained. Early stopping was applied when validation discrimination failed to improve, while the independent 70-patient testing subset remained unused during optimization and model selection.

Training augmentation was applied only to the internal training subset and was performed in a paired manner to preserve the spatial correspondence between T0 and T1. For the compact custom CNN, paired horizontal and vertical flips were each applied with a probability of 0.5. For the paired ResNet18 experiment, horizontal flips were applied with a probability of 0.5, vertical flips with a probability of 0.3, and low-amplitude Gaussian noise with a standard deviation of 0.01 with a probability of 0.3. Identical geometric transformations were applied to both longitudinal inputs. No augmentation was applied to the validation or independent test subsets.

Because the present study involved a relatively small longitudinal imaging cohort, this end-to-end branch was treated explicitly as exploratory. Its role within the study design was to test whether direct paired-image modeling was feasible and whether it could generate a stable predictive signal under the current sample-size constraints. As described in the Results, both the compact custom CNN and the partially fine-tuned ResNet18 architecture demonstrated marked divergence between training and validation performance. Thus, the end-to-end branch was retained as an explicit feasibility and sensitivity analysis but was not selected as the principal deep representation strategy. A subsequent frozen pretrained feature-extraction approach was therefore evaluated to reduce the instability associated with direct end-to-end optimization.

### 2.16. Frozen Pretrained ResNet18 Embedding Extraction

Because the exploratory end-to-end paired deep learning experiments showed substantial overfitting under the present sample-size constraints, a second image-based strategy was implemented in which a pretrained convolutional backbone was used only as a fixed feature extractor rather than as a fully trainable predictive model. The purpose of this branch was to obtain deep image-derived latent representations from the paired T0 and T1 tensors while limiting the instability associated with end-to-end optimization in a modest longitudinal cohort.

For this purpose, an ImageNet-pretrained ResNet18 architecture was adopted as the backbone network [35,36,37]. The original first convolutional layer, which used pretrained weights with dimensions 64 × 3 × 7 × 7 for conventional three-channel images, was replaced by a seven-channel convolutional layer with dimensions 64 × 7 × 7 × 7. To preserve the ImageNet initialization, the pretrained convolutional filters were averaged across the original RGB input-channel dimension, producing a single mean filter for each output channel, and the resulting filters were repeated across the seven input channels. The same adapted ResNet18 backbone, with identical shared weights, was applied to both the T0 and T1 tensors. All backbone parameters were then frozen. The final fully connected classification layer was replaced by an identity mapping, so that global average pooling produced one 512-dimensional latent feature vector for each input tensor.

The adapted ResNet18 was then used in frozen mode, with weights held fixed during embedding extraction. For each patient, the T0 tensor and the T1 tensor were passed independently through the pretrained backbone to generate two latent feature vectors, one representing the baseline lesion appearance and one representing the early-treatment lesion appearance. In addition to these direct embeddings, two derived deep-representation forms were computed: a delta embedding, defined as the element-wise difference between the T1 and T0 latent vectors, and an average embedding, defined as the element-wise mean of the T0 and T1 latent vectors. This produced four deep representation blocks for each patient: T0, T1, DELTA, and AVG.

Each representation block had dimensionality 512, corresponding to the output size of the frozen ResNet18 backbone after global pooling. The resulting patient-level embedding table therefore contained four 512-dimensional blocks, yielding a total of 2048 deep image-derived features per patient, in addition to patient identifier, predefined split assignment, and pCR label. Embedding extraction was applied to the full 183-patient cohort, preserving the predefined BMMR2 split structure of 113 training patients and 70 testing patients.

The rationale for including both direct and derived embedding forms was to evaluate whether deep image representations of absolute lesion state and deep image representations of longitudinal change carried different predictive information. In this way, the frozen embedding branch provided a deep representation analogue of the handcrafted T0, T1, and delta radiomic feature blocks, while avoiding the strong overfitting tendency observed in the end-to-end paired deep learning experiments.

ImageNet-pretrained ResNet18 was selected as a widely available, reproducible, and comparatively compact reference backbone [35,36,37]. Its comparatively modest architectural complexity and parameter count were considered appropriate for frozen feature extraction in a development cohort of only 113 patients. The purpose of this branch was to compare frozen latent image representations with handcrafted radiomics rather than to conduct an exhaustive architecture benchmark. Newer pretrained architectures were not included because comparison of multiple backbone families, architectural scales, and pretraining strategies would introduce an additional model-selection dimension and could increase the risk of optimistic architecture selection within the same limited cohort. Medical-domain pretraining, including RadImageNet-based architectures, may improve transferability and should be evaluated in future multicohort studies.

### 2.17. Predictive Modeling on Frozen Deep Image Embeddings

After extraction of the frozen pretrained image embeddings, predictive modeling was performed on the resulting patient-level deep representation table in order to evaluate whether latent image features derived from the paired T0 and T1 tensors could predict pathologic complete response (pCR). As in the handcrafted radiomics branch, the predefined BMMR2 split structure was preserved throughout this stage, such that the 113-patient training subset was used for model development and the 70-patient testing subset was reserved for independent performance evaluation.

Several embedding-based representation blocks were evaluated separately and in combination. These included: (i) baseline T0 embeddings alone; (ii) early-treatment T1 embeddings alone; (iii) delta embeddings alone, defined as the element-wise difference between T1 and T0 latent vectors; (iv) average embeddings alone, defined as the element-wise mean of the T0 and T1 latent vectors; (v) combined T0 and T1 embeddings; (vi) combined T1 and delta embeddings; and (vii) the full integrated embedding table containing T0, T1, delta, and average representations together. This design was chosen in order to determine whether the most informative deep representation was linked primarily to baseline lesion appearance, early-treatment lesion appearance, longitudinal change, or integrated paired-image information.

Before classifier fitting, train-only filtering and ranking procedures were applied within each candidate embedding block. Features with no usable variation in the training subset were excluded, and highly correlated variables were removed on the basis of training-set correlation structure alone. The remaining candidate embedding features were then ranked using training-only relevance scoring, and compact feature budgets were evaluated in order to limit redundancy and reduce the risk of overfitting. This yielded a staged small-sample modeling procedure analogous in spirit to the refined handcrafted radiomics branch, but applied to latent image-derived features rather than engineered descriptors.

Downstream predictive modeling was then performed using a set of complementary classifiers representing both linear and non-linear learning strategies. These included logistic regression, random forest, and gradient-boosted tree methods. For the frozen-embedding analyses, logistic regression was implemented after median imputation and standard scaling using C = 0.1, balanced class weights, a maximum of 5000 iterations, and a random seed of 42. Random forest used median imputation, 400 trees, a maximum depth of 5, a minimum terminal-leaf size of 3, square-root feature sampling, balanced class weights, and a random seed of 42. XGBoost used median imputation, 300 estimators, a maximum depth of 3, a learning rate of 0.03, subsampling and column-subsampling fractions of 0.8, L2 regularization of 1.0, and a binary logistic objective. Within each embedding block, features were ranked using training-only mutual information after correlation pruning at an absolute threshold of 0.95, and feature budgets of 10, 20, 30, 50, and 100 were evaluated. Within each model, missing values were handled using training-derived imputation only, and any scaling steps required by the classifier were fitted exclusively on the training subset and then applied unchanged to the testing subset. This ensured that no information from the held-out testing cohort influenced feature preprocessing, feature ranking, or model fitting.

Predictive performance was evaluated primarily on the independent testing subset using the same discrimination framework as in the handcrafted radiomics analyses. The principal evaluation metrics were the area under the receiver operating characteristic curve (AUROC) and the area under the precision–recall curve (AUPRC). Additional operating-characteristic measures included accuracy, sensitivity, specificity, precision, F1 score, and confusion-matrix-derived counts. The objective of this stage was not only to identify the strongest deep embedding configuration, but also to compare the behavior of frozen pretrained image representations against the handcrafted radiomics benchmark within the same longitudinal cohort and fixed train/test evaluation design.

For the principal retained handcrafted and embedding-based models, uncertainty of AUROC and AUPRC on the independent testing subset was estimated using stratified bootstrap resampling with 2000 replicates, and 95% confidence intervals were derived using the percentile method. Because the strongest handcrafted radiomics model and the strongest frozen deep-embedding model were evaluated on the same independent test patients, their AUROC values were compared using paired resampling procedures. The primary comparison involved the T1-only 10-feature radiomics random forest model and the DELTA-only 30-feature frozen-embedding random forest model. The confidence interval for the paired AUROC difference was estimated using 10,000 stratified paired-bootstrap replicates, preserving the pCR and non-pCR class counts within each replicate. A two-sided paired permutation test with 10,000 repetitions was additionally used to test the null hypothesis of no difference in AUROC between the two models. The same stratified paired-bootstrap procedure was used to estimate the 95% confidence interval for the paired difference in AUPRC.

Calibration was evaluated descriptively for the two models included in the primary paired comparison using their unchanged predicted probabilities on the predefined 70-patient held-out benchmark subset. Overall probabilistic accuracy was summarized using the Brier score. Calibration-in-the-large and probability dispersion were assessed using the calibration intercept and calibration slope obtained from logistic recalibration of the observed outcome on the logit of the predicted probability. These estimates were used only for evaluation, and no post hoc recalibration was applied to the reported predictions. Calibration curves were constructed using five quantile-based groups of predicted probabilities. Because the benchmark subset contained only 70 patients, all calibration estimates were considered exploratory.

All data-dependent preprocessing and model-fitting procedures, including imputation, scaling, feature filtering, correlation pruning, mutual-information ranking, hyperparameter optimization, checkpoint selection, and probability-threshold selection, were performed without access to the testing labels. However, several candidate representation blocks, feature budgets, and classifier configurations were evaluated on the same predefined held-out testing subset. Consequently, this subset functioned as a common comparative benchmark rather than as a single-use confirmatory validation cohort. The terms “strongest” and “retained” refer to the configurations achieving the highest observed benchmark AUROC or AUPRC within their respective analysis branches. Because repeated comparative evaluation may introduce model-comparison optimism, all reported benchmark results were interpreted as exploratory.

## 3. Results

### 3.1. First-Pass Predictive Benchmarking of Clinical and Radiomic Feature Blocks

A first-pass predictive benchmark was performed using the cleaned machine-learning-ready matrix derived from the 183-patient cohort, preserving the predefined split of 113 training patients and 70 testing patients. Multiple feature representations were compared, including clinical-only, T0-only, T1-only, delta-only, combined T0 + T1 radiomics, combined T0 + T1 + delta radiomics, and clinical plus all imaging features. For each representation, logistic regression, random forest, and gradient-boosted tree models were evaluated on the predefined held-out benchmark subset. Table 2 summarizes first-pass predictive performance on the independent testing subset across the evaluated clinical, radiomic, and longitudinal feature representations.

The zero precision, sensitivity, and F1 values of the two delta-only first-pass models were verified against their confusion-matrix counts. The random forest model produced TN = 49, FP = 1, FN = 20, and TP = 0, whereas the gradient-boosted model produced TN = 47, FP = 3, FN = 20, and TP = 0. The zero-valued metrics therefore reflect genuine operating-point behavior at the prespecified threshold and not a division-by-zero or implementation error.

Overall, predictive performance in this initial benchmark was modest across all evaluated configurations. The best-performing model was the clinical-only logistic regression model, which achieved an AUROC of 0.628 and an AUPRC of 0.395 on the independent test subset. Among the imaging-based models, the strongest result was obtained with the combined T0 + T1 radiomic representation using random forest, which achieved an AUROC of 0.623. T0-only and T1-only radiomic models showed similar but slightly lower performance, whereas the delta-only feature block performed more weakly and did not provide competitive discrimination in this first-pass setting.

Expansion to higher-dimensional combined feature spaces, including T0 + T1 + delta radiomics and clinical plus all imaging variables, did not improve performance relative to the simpler baseline configurations. In particular, the larger integrated models tended to preserve relatively high specificity but showed limited sensitivity, indicating that the raw high-dimensional feature space may have introduced redundancy and diluted predictive signal. Thus, although radiomic information was not uninformative, the unfiltered first-pass radiomic representations did not outperform the compact clinical baseline.

These findings support two conclusions. First, the full longitudinal pipeline—from public archive preprocessing to radiomic extraction, longitudinal feature construction, and train/test modeling—was operationally successful. Second, the modest performance of the raw merged radiomic feature space indicates that additional train-only feature selection and dimensionality reduction are necessary before final comparative modeling and explainable analysis.

### 3.2. Implication of the First-Pass Benchmark for Subsequent Modeling

Because the first-pass models used the cleaned but otherwise largely unfiltered feature matrix, the resulting benchmark should be interpreted as an initial screening analysis rather than a final optimized performance estimate. The fact that the best imaging-based models approached but did not exceed the clinical-only baseline suggests that relevant radiomic signal may be present but partially obscured by dimensionality, redundancy, and residual non-informative variation. Accordingly, the next analytical step was defined as train-only feature selection and refinement of the predictive models before subsequent explainable AI interpretation. Because the subsequent refinement stage was informed by the comparative performance observed in the predefined benchmark subset, this staged analysis was treated as exploratory rather than confirmatory.

### 3.3. Feature-Selected Refinement of the Strongest Radiomic Blocks

Because the first-pass benchmark suggested that the raw merged radiomic feature space remained overly high-dimensional relative to the size of the training subset, a second modeling stage was performed using train-only feature selection and correlation pruning. Within each candidate feature block, variables with no usable variation in the training subset were excluded, highly correlated features were removed, and the remaining predictors were ranked using training-only relevance scoring. Compact feature budgets were then evaluated in order to reduce redundancy and improve signal concentration before classifier fitting.

This refinement stage improved predictive discrimination relative to the initial unfiltered benchmark. The best-performing configuration was obtained using the T1-only radiomic representation with random forest and a top-10 selected feature set, yielding an AUROC of 0.670 (95% CI, 0.513–0.811) and an AUPRC of 0.405 (95% CI, 0.319–0.607) on the independent testing subset. A closely related configuration based on the combined T0 + T1 radiomic representation with random forest and a top-20 selected feature set achieved an AUROC of 0.665 and an AUPRC of 0.394. Both results exceeded the performance of the compact clinical baseline observed in the first-pass benchmark, which had achieved an AUROC of 0.628.

Inspection of the selected predictors showed that the refined models were driven predominantly by biologically plausible imaging descriptors rather than technical or administrative variables. In particular, the strongest T1-only model emphasized lesion size, lesion morphology, shape anisotropy, statistical dispersion, and texture non-uniformity at the early-treatment timepoint. The combined T0 + T1 models additionally incorporated baseline texture and morphology descriptors. By contrast, longitudinal delta-only representations remained less competitive, suggesting that the early-treatment radiomic phenotype may currently provide a stronger predictive signal than longitudinal difference features alone.

Although this refinement stage improved performance relative to the first-pass benchmark, overall discrimination remained modest. A subsequent targeted tuning stage focused only on the strongest feature blocks, namely T1-only, T0 + T1, and clinical + T0 + T1. This additional tuning improved cross-validated training performance but did not improve discrimination on the independent testing subset. As a secondary operating-threshold sensitivity analysis, probability thresholds were selected exclusively from out-of-fold training predictions. For the highest-ranking tuned T1-only random forest configuration, based on eight selected features, the train-only Youden procedure selected a probability threshold of 0.38. On the independent test subset, reducing the threshold from 0.50 to 0.38 increased sensitivity from 0.25 to 0.50, accuracy from 0.643 to 0.714, and the F1 score from 0.286 to 0.500, while specificity remained 0.80. AUROC and AUPRC remained 0.639 and 0.410, respectively, because these rank-based metrics are independent of the classification threshold. The complete comparison is reported in Table S1. Although threshold adjustment improved the operating-point balance, it did not improve rank discrimination relative to the retained primary T1-only random forest model, which achieved an AUROC of 0.670. Accordingly, the earlier feature-selected T1-only random forest model with 10 selected features was retained as the strongest handcrafted imaging configuration. Thus, within the handcrafted radiomics branch, the clearest predictive signal was concentrated in the early-treatment T1 phenotype rather than in baseline-only or simple longitudinal delta representations.

Table 3 summarizes the strongest feature-selected configurations obtained after train-only filtering, correlation pruning, and compact-feature-budget modeling.

After train-only feature selection, the strongest performance was achieved by the T1-only radiomic model, indicating that the early-treatment T1 phenotype carried the clearest handcrafted predictive signal in the present dataset.

### 3.4. Composition of the Winning T1 Feature Set

To better understand the source of the strongest imaging-based signal, the 10 radiomic features retained in the selected T1-only random forest model were examined. These predictors were dominated by lesion size and morphology descriptors, together with first-order dispersion measures and run-length texture heterogeneity terms. Table 4 lists the selected T1 features and their approximate imaging interpretation.

Overall, the retained T1 feature set suggests that early-treatment predictive information in the present cohort is carried mainly by lesion burden, geometric structure, shape anisotropy, intensity dispersion, and texture non-uniformity rather than by a single isolated descriptor. This pattern is consistent with the broader observation that the early-treatment T1 phenotype provided the clearest imaging-based signal in the refined benchmark, although the resulting discrimination remained modest and should therefore be interpreted as exploratory rather than definitive.

**Figure 2:**
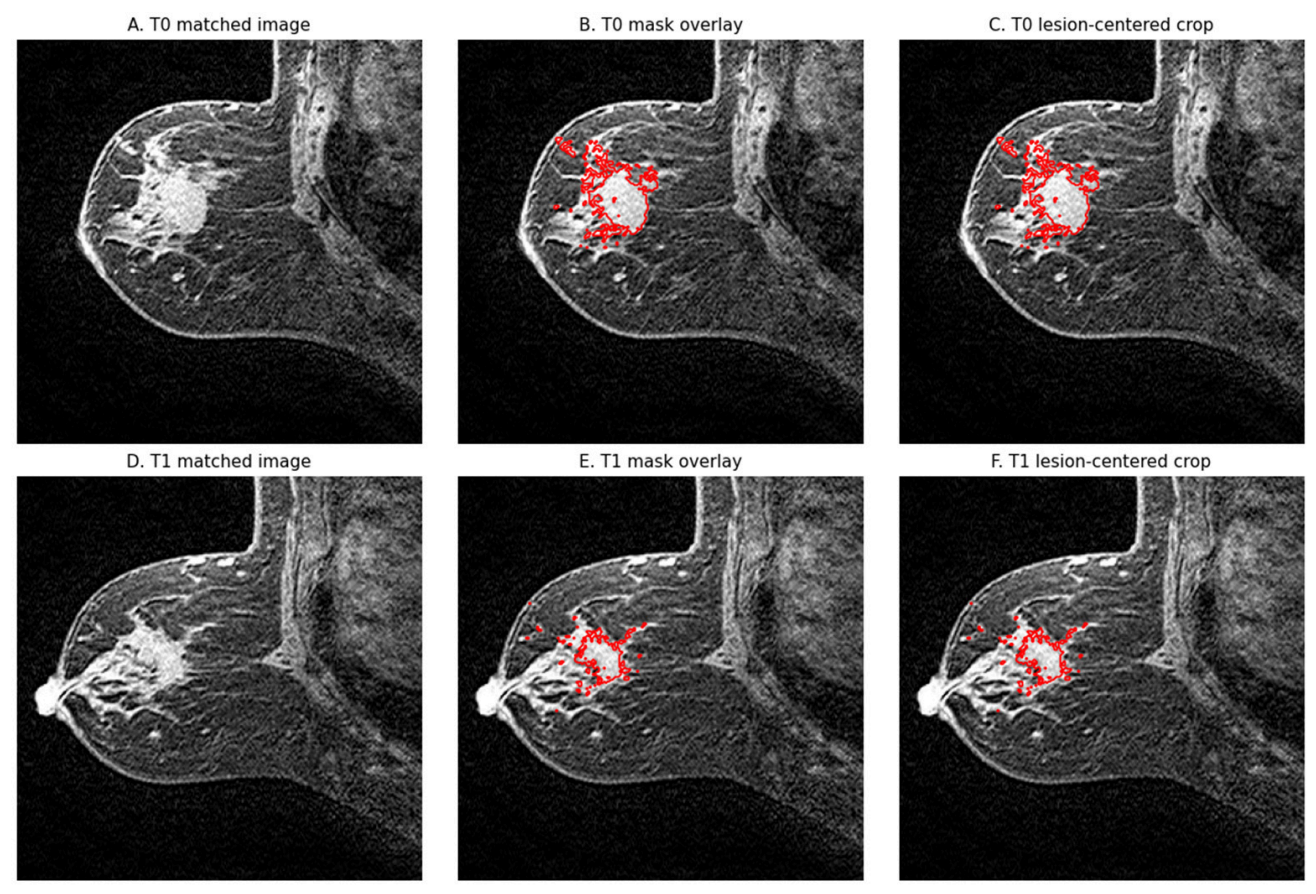
Representative matched longitudinal case and lesion-centered reconstruction from the final 183-patient cohort. Panels (**A**–**C**) show the T0 matched image, lesion-mask overlay, and lesion-centered crop, respectively. Panels (**D**–**F**) show the corresponding T1 matched image, lesion-mask overlay, and lesion-centered crop. This representative example illustrates the validity of the phase-matching and segmentation-decoding workflow and demonstrates the visual basis for both handcrafted radiomics and deep image-based modeling.

Representative examples of the matched longitudinal image–mask reconstruction are shown in Figure 2. The figure illustrates a typical patient-level T0/T1 pair after phase matching and lesion-mask decoding, confirming visually that the selected MR phase and corresponding lesion ROI were spatially coherent and suitable for downstream radiomic extraction and lesion-centered deep-learning dataset construction.

### 3.5. Exploratory End-to-End Paired T0/T1 Deep Learning Performance

To determine whether treatment-response information could be learned directly from paired longitudinal image tensors rather than from predefined handcrafted descriptors, exploratory end-to-end deep learning experiments were performed using the lesion-centered paired T0/T1 2.5D dataset described in Section 2.14. In these experiments, image-only paired models were trained directly on the T0 and T1 inputs, without handcrafted radiomic features, using the predefined 113-patient training cohort for model development and the independent 70-patient testing cohort for final assessment.

**Figure 3:**
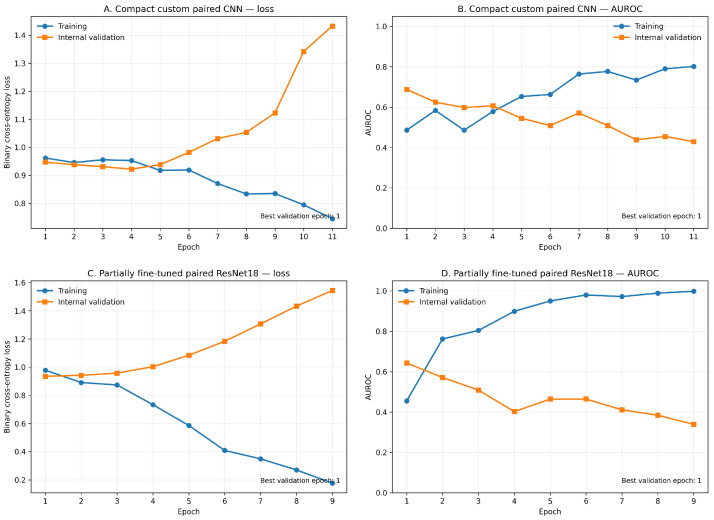
Training dynamics of the exploratory end-to-end paired T0/T1 models. Panels (**A**,**B**) show the training and internal-validation loss and AUROC, respectively, for the compact custom shared-weight CNN. Panels (**C**,**D**) show the corresponding training dynamics for the partially fine-tuned paired ResNet18 architecture, in which Layer 4 and the fusion head were trainable. In both experiments, the highest validation AUROC occurred at epoch 1, followed by progressive divergence between training and validation behavior, indicating marked overfitting.

Both exploratory architectures were technically feasible and generated valid patient-level probabilistic outputs on the independent test subset. However, neither achieved stable generalization. The compact custom shared-weight CNN reached its highest internal-validation AUROC of 0.688 at epoch 1. During subsequent epochs, training discrimination continued to improve, whereas validation loss increased and validation discrimination became unstable. Early stopping occurred at epoch 11, and the checkpoint corresponding to epoch 1 was retained. The corresponding training and internal-validation loss and AUROC trajectories are shown in Figure 3A,B. On the independent test subset, this model achieved an AUROC of 0.433, an AUPRC of 0.256, and an accuracy of 0.286. At the prespecified probability threshold of 0.5, the model classified all test cases as pCR, resulting in TN = 0, FP = 50, FN = 0, and TP = 20, with sensitivity of 1.00, specificity of 0.00, and an F1 score of 0.444.

Partial fine-tuning of the paired pretrained ResNet18 architecture showed a similar but opposite operating-point instability. The highest internal-validation AUROC was 0.643 at epoch 1. Training AUROC subsequently increased to 0.998, whereas validation AUROC deteriorated to 0.339, and training was stopped at epoch 9. The corresponding training and internal-validation trajectories are shown in Figure 3C,D. The retained epoch-1 checkpoint achieved an AUROC of 0.504, an AUPRC of 0.353, and an accuracy of 0.714 on the independent test subset. At the threshold of 0.5, this model classified all test cases as non-pCR, resulting in TN = 50, FP = 0, FN = 20, and TP = 0, with sensitivity of 0.00, specificity of 1.00, and an F1 score of 0.000.

The two exploratory architectures therefore exhibited different degenerate operating-point behaviors despite the use of class-weighted loss, paired augmentation, weight decay, and early stopping. The compact custom CNN favored the positive class almost exclusively, whereas the partially fine-tuned ResNet18 favored the negative class exclusively at the prespecified threshold. These results indicate that reducing architectural complexity or partially fine-tuning the final pretrained block did not resolve the instability caused by the limited development cohort. The reported zero-valued sensitivity, specificity, or F1 measures reflect the actual confusion-matrix behavior of the models and not a numerical failure in metric calculation. The quantitative validation and independent test-set performance of both exploratory architectures are summarized in Table 5.

Because both exploratory models selected their strongest validation checkpoint during the first training epoch and subsequently showed pronounced training–validation divergence, the end-to-end branch was interpreted as a feasibility and sensitivity analysis rather than as the principal deep-learning benchmark. Importantly, the experiments included both a compact custom CNN and partial fine-tuning of the final ResNet18 block, but neither strategy provided stable test-set discrimination. Subsequent deep image analysis was therefore redirected toward frozen pretrained feature extraction, which substantially reduced the number of trainable parameters and enabled a more stable comparison with the handcrafted radiomics branch.

### 3.6. Performance of Frozen Pretrained Image Embeddings

Because the exploratory end-to-end paired-image models showed substantial overfitting, a frozen pretrained feature-extraction strategy was adopted as the principal deep image representation branch. Using the paired T0/T1 2.5D tensors, patient-level latent image representations were extracted with a frozen ResNet18 backbone, yielding T0, T1, DELTA, and AVG embedding blocks as described in Section 2.16. Downstream predictive models were then trained on train-only filtered and ranked embedding subsets using the predefined 113-patient training cohort and evaluated on the independent 70-patient testing cohort.

The strongest embedding-based results are summarized in Table 6. Across the evaluated representation blocks, predictive performance remained modest but was comparable to that obtained with the strongest handcrafted radiomics models. The best-performing embedding configuration by AUROC was obtained using the DELTA-only representation with random forest and a 30-feature budget, yielding an AUROC of 0.672 (95% CI, 0.529–0.812) and an AUPRC of 0.408 (95% CI, 0.314–0.610) on the independent testing subset. A closely related DELTA-only configuration using gradient-boosted trees and a 50-feature budget yielded an AUROC of 0.666 and an AUPRC of 0.393.

When the evaluation was viewed from the perspective of precision–recall performance, the strongest embedding-based result was obtained using the AVG-only representation with logistic regression and a 100-feature budget, yielding an AUROC of 0.666 (95% CI, 0.529–0.806) and an AUPRC of 0.472 (95% CI, 0.343–0.671). This configuration also showed a more balanced operating behavior than several of the higher-specificity embedding models, with sensitivity of 0.55, specificity of 0.74, and F1 score of 0.50. Thus, although the best embedding-based AUROC and the best embedding-based AUPRC were achieved by different representation–classifier combinations, both results supported the conclusion that frozen pretrained deep image representations carried meaningful but still exploratory predictive signal in the present cohort.

The key pattern in Table 6 is that the strongest frozen deep image-embedding signal was not concentrated in the absolute T1 embedding alone, but in derived longitudinal latent representations. Specifically, the best embedding-based result by AUROC was obtained from the DELTA-only representation, whereas the best embedding-based result by AUPRC was obtained from the AVG-only representation. This indicates that the frozen embedding branch was stable and informative, but that its most useful predictive content was carried by latent change-oriented and integrated paired-image representations rather than by the absolute early-treatment state alone.

At the same time, overall discrimination remained modest, and none of the frozen embedding configurations achieved strong clinical-grade predictive performance. The appropriate interpretation is therefore not that frozen deep image embeddings solved the pCR prediction problem in this cohort, but rather that they provided a stable, competitive, and methodologically informative comparator after the exploratory end-to-end paired-image models had overfit. Thus, within the retained deep image branch, the principal signal was concentrated in DELTA for AUROC and in AVG for AUPRC.

### 3.7. Comparison Between Handcrafted Radiomics and Frozen Deep Image Embeddings

To place the frozen image-embedding results in context, the strongest deep representation models were compared directly with the strongest handcrafted radiomics benchmark. The corresponding results are summarized in Table 7. In the handcrafted radiomics branch, the best-performing refined configuration was the T1-only random forest model with a 10-feature budget, which achieved an AUROC of 0.670 (95% CI, 0.513–0.811) and an AUPRC of 0.405 (95% CI, 0.319–0.607) on the independent testing subset. In the frozen deep-embedding branch, the best-performing configuration by AUROC was the DELTA-only random forest model with a 30-feature budget, which achieved an AUROC of 0.672 (95% CI, 0.529–0.812) and an AUPRC of 0.408 (95% CI, 0.314–0.610). The strongest embedding configuration by AUPRC was the AVG-only logistic regression model with a 100-feature budget, which achieved an AUROC of 0.666 (95% CI, 0.529–0.806) and an AUPRC of 0.472 (95% CI, 0.343–0.671).

A formal paired comparison was performed between the strongest handcrafted radiomics model and the strongest frozen deep-embedding model by AUROC. The DELTA-only frozen-embedding random forest model achieved an AUROC 0.002 higher than that of the T1-only radiomics random forest model. The stratified paired-bootstrap 95% confidence interval for this difference ranged from −0.179 to 0.184, and the two-sided paired permutation test yielded *p* = 0.988. The corresponding paired difference in AUPRC was 0.003, with a 95% confidence interval ranging from −0.201 to 0.211. These results provide no evidence of a statistically significant difference in discrimination between the two models in the present test cohort.

**Figure 4:**
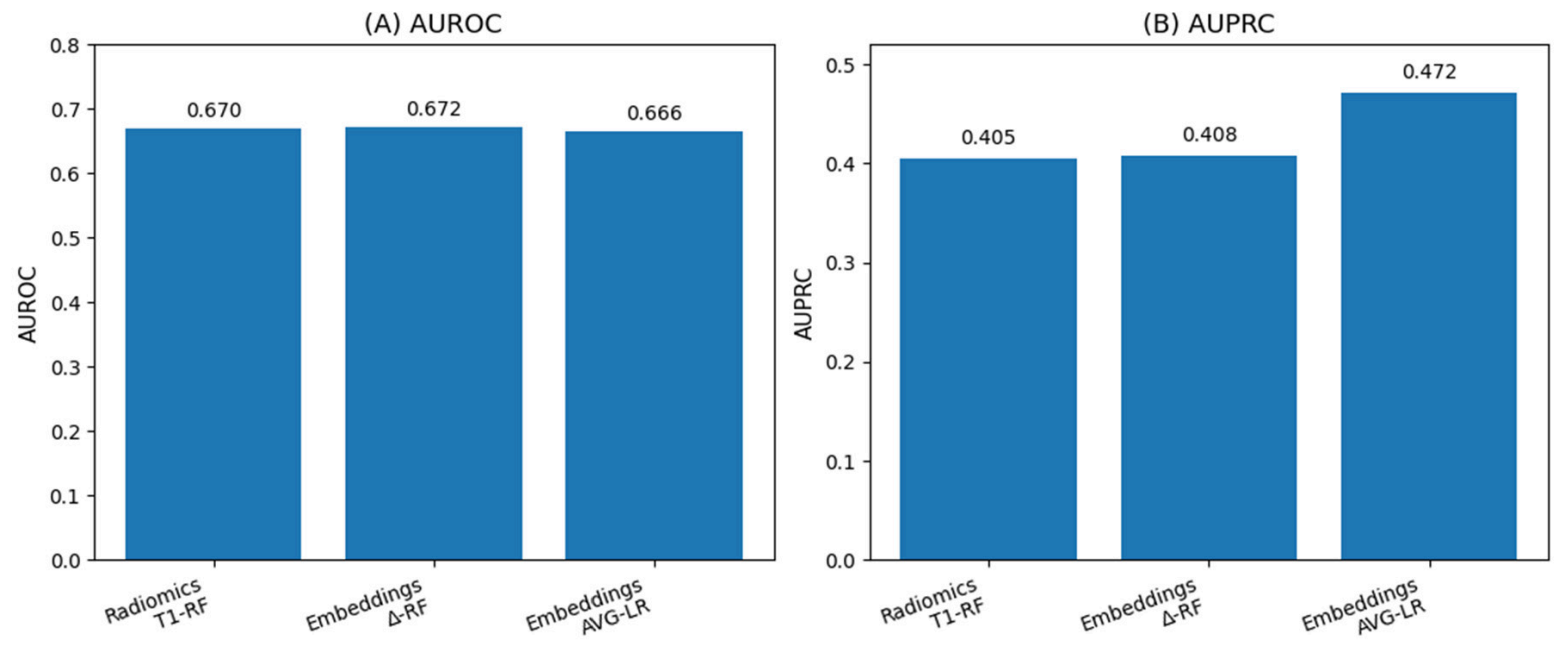
Comparative test-set discrimination of the strongest handcrafted radiomics and frozen deep image-embedding models. Panel (**A**) compares AUROC values and panel (**B**) compares AUPRC values for the strongest retained handcrafted radiomics model and the strongest retained frozen deep image-embedding models. AUROC values were similar across the retained models, whereas the AVG-only frozen embedding logistic regression model yielded the highest AUPRC, indicating that handcrafted radiomics and frozen deep embeddings captured partly different aspects of early treatment response.

The formal paired comparison, together with the overlapping individual confidence intervals, supports the interpretation that the retained handcrafted radiomics and frozen deep-embedding models achieved statistically comparable discrimination in the present exploratory cohort. To facilitate direct visual comparison between the strongest handcrafted radiomics model and the strongest frozen deep image-embedding models, their principal test-set discrimination metrics are summarized in Figure 4.

As shown in Figure 4, AUROC values were closely similar across the strongest retained radiomics and embedding-based models, and the corresponding confidence intervals overlapped substantially. However, the AVG-only frozen embedding logistic regression model achieved the highest AUPRC, suggesting that the frozen deep representation may better prioritize positive-response cases under class imbalance, even though its overall rank discrimination remained broadly comparable to that of the strongest handcrafted radiomics model.

Several points emerge from this comparison. First, the strongest handcrafted and frozen deep representation models achieved closely similar overall performance levels. Thus, the deep image-embedding branch did not provide a major absolute performance gain over the refined handcrafted radiomics benchmark in the present cohort. The main value of the embedding branch therefore lies less in dramatic predictive improvement than in the complementary representational perspective it provides.

Second, the strongest signal was concentrated in different feature blocks across the two representation families, and this contrast is the main conceptual result of the comparison. In the handcrafted radiomics branch, the most informative model was based on the absolute early-treatment T1 phenotype. In the frozen deep-embedding branch, the strongest AUROC was obtained from the DELTA-only latent representation, whereas the strongest AUPRC was obtained from the AVG-only latent representation. This pattern suggests that handcrafted descriptors and pretrained latent image features were not redundant summaries of the same lesion information, but instead emphasized different aspects of treatment response.

Third, the comparison indicates that the retained deep representation branch is most informative when interpreted as a complementary view of longitudinal change rather than as a replacement for handcrafted radiomics. The engineered radiomic features appeared to emphasize the lesion state observed at the early-treatment timepoint, whereas the frozen deep image embeddings concentrated their strongest signal in latent longitudinal change and integrated paired-image state. Accordingly, the comparative analysis supports the interpretation that early treatment response may be represented differently in handcrafted and deep latent feature spaces, and that this representational complementarity is more important than any small difference in headline AUROC alone.

**Figure 5:**
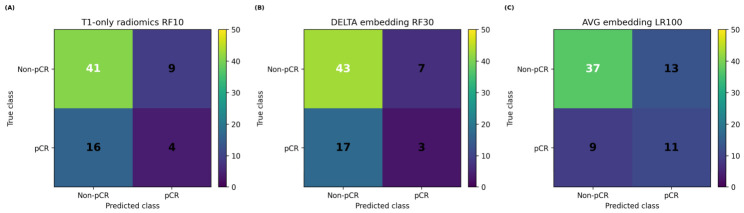
Confusion matrices of the principal retained models on the independent 70-patient test subset at the prespecified probability threshold of 0.5. Panel (**A**) shows the T1-only 10-feature radiomics random forest model, which produced TN = 41, FP = 9, FN = 16, and TP = 4. Panel (**B**) shows the DELTA-only 30-feature frozen-embedding random forest model, which produced TN = 43, FP = 7, FN = 17, and TP = 3. Panel (**C**) shows the AVG-only 100-feature frozen-embedding logistic regression model, which produced TN = 37, FP = 13, FN = 9, and TP = 11. The AVG embedding model provided the most balanced sensitivity–specificity profile, whereas the T1 radiomics and DELTA embedding models favored specificity.

To complement the aggregate discrimination and operating-point metrics, confusion matrices for the three principal retained models are presented in Figure 5. The T1-only radiomics and DELTA embedding models predominantly favored the non-pCR class and therefore achieved relatively high specificity but low sensitivity. By contrast, the AVG embedding logistic regression model provided a more balanced distribution of true-positive and true-negative classifications.

**Figure 6:**
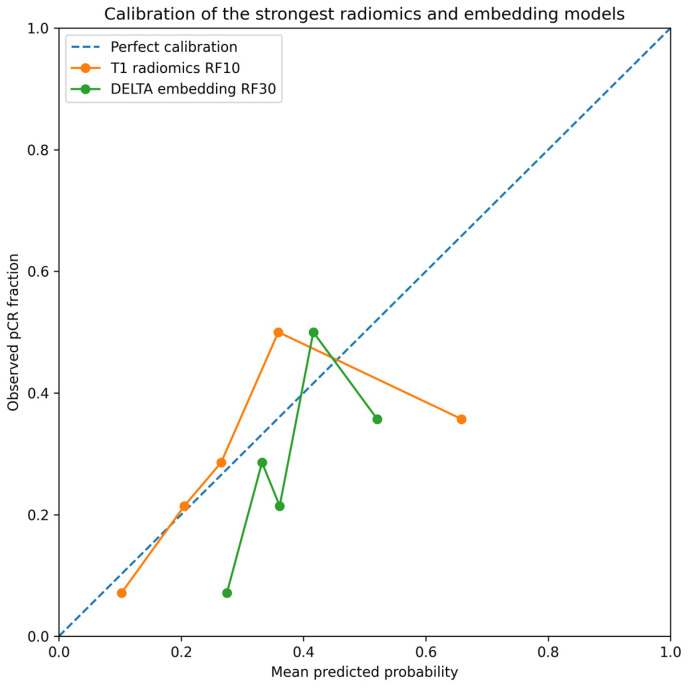
Calibration curves for the strongest handcrafted radiomics and frozen deep-embedding models on the predefined 70-patient held-out benchmark subset. The observed pCR fraction is plotted against the mean predicted probability across five quantile-based probability groups. The diagonal line represents perfect calibration. Calibration estimates are exploratory because of the limited benchmark-subset size.

Calibration was additionally assessed for the two models included in the primary paired comparison. The summary calibration metrics are presented in Table 8. The corresponding observed-versus-predicted probability relationships are shown in Figure 6, while the numerical values for the five quantile-based probability groups used to construct the curves are provided in Table S3. The T1-only radiomics random forest model achieved a Brier score of 0.211, a calibration intercept of −0.569, and a calibration slope of 0.428. The DELTA-only frozen-embedding random forest model achieved a Brier score of 0.204, a calibration intercept of −0.294, and a calibration slope of 1.340. The observed pCR prevalence was 0.286, whereas the mean predicted probabilities were 0.318 and 0.381, respectively. These findings indicate imperfect probability calibration despite the similar discrimination of the two models. Given the limited 70-patient benchmark subset, the calibration estimates should be interpreted as exploratory rather than as evidence of transportable individual-risk estimation.

The calibration curves corresponding to the two models are presented in Figure 6.

## 4. Discussion

The present study set out to determine whether a large public longitudinal breast MRI archive could be transformed into a reproducible, analysis-ready cohort suitable for early treatment-response modeling, and whether both handcrafted radiomic descriptors and image-derived deep representations obtained from this pipeline could provide predictive information for pathologic complete response (pCR). Several points emerge from the results.

First, the study demonstrates that the end-to-end workflow was operationally successful. Starting from the large and heterogeneous ACRIN 6698/BMMR2 public archive, the preprocessing pipeline reduced the parent dataset to a fully matched 183-patient longitudinal imaging cohort with validated T0 and T1 phase-matched image–mask pairs, preserved train/test assignment, binary pCR labeling, cohort-wide radiomic extraction, and paired lesion-centered image tensors suitable for downstream deep representation learning. This is an important result in itself, because the raw archive was not immediately ready for direct longitudinal quantitative analysis. Considerable preprocessing was required to harmonize clinical metadata, map DICOM inventory to clinical records, identify the correct cropped-DCE branch, recover usable T0/T1 candidate studies, resolve the multi-phase DCE structure, decode segmentation objects, reconstruct matched image–mask pairs suitable for radiomics, and convert the resulting data into a standardized 2.5D paired image representation. Thus, beyond the predictive modeling results, the study provides a practical and reproducible framework for converting a complex public oncologic MRI archive into a structured longitudinal modeling resource.

Second, the initial predictive benchmark showed that the cleaned, but otherwise largely unfiltered radiomic space was too high-dimensional relative to the size of the training subset. In the first-pass analysis, the compact clinical-only logistic regression model slightly outperformed the raw radiomic configurations, while the larger combined imaging blocks failed to improve discrimination. This pattern strongly suggested that the merged radiomic feature space still contained substantial redundancy and non-informative variation. The fact that larger blocks did not help, and sometimes appeared to worsen sensitivity despite reasonable specificity, is consistent with the known instability of high-dimensional radiomics modeling in modest sample-size settings. In other words, the initial weak-to-modest performance did not imply that MRI-derived information was absent, but rather that the informative signal was diluted by feature burden.

Third, train-only feature selection materially changed the picture. After correlation pruning and compact feature-budget modeling, the strongest imaging-based handcrafted result was obtained from the T1-only radiomic representation, whereas raw delta-only models remained less competitive. This is one of the most interesting findings of the study. It suggests that, in the present cohort, the early-treatment lesion phenotype captured at T1 may contain more immediately usable predictive information than either baseline T0 descriptors alone or unfiltered longitudinal difference features alone. The selected T1 predictors were dominated by lesion size, morphology, shape anisotropy, first-order dispersion, and run-length texture heterogeneity. This is biologically plausible. Residual tumor burden, residual geometric complexity, and residual heterogeneity after early treatment exposure may reflect incomplete therapeutic response more directly than baseline lesion appearance in isolation. Conversely, the weaker standalone performance of delta-only handcrafted features may indicate that simple arithmetic change descriptors do not yet capture treatment evolution in the most informative way, at least within the present feature construction strategy. This pattern is compatible with prior MRI studies showing that both early-treatment lesion phenotype and explicitly longitudinal imaging change can carry response-related information, although the optimal representation may depend strongly on cohort composition, imaging protocol, and feature-construction strategy [14,15,24,25].

At the same time, the overall predictive discrimination remained modest. Even after train-only selection, the best-performing T1-based handcrafted model achieved only exploratory-level performance rather than strong clinical-grade discrimination. This is an important limitation of interpretation. The current results support the claim that early-treatment MRI-derived information contains predictive signal, but they do not support a claim of highly accurate standalone pCR prediction. In this sense, the study should be understood as a proof-of-workflow and proof-of-signal analysis rather than as a definitive predictive solution. The appropriate scientific message is therefore more modest but still meaningful: early-treatment lesion phenotype appears more informative than the raw full merged feature space, and careful train-only filtering and selection are necessary to reveal that signal. This interpretation is also consistent with recent systematic reviews and meta-analyses indicating that MRI-based radiomics and machine-learning models in the neoadjuvant breast cancer setting frequently show encouraging but still heterogeneous and methodologically variable performance, with continued need for standardization and external validation [4,5,6,28].

The present results should also be interpreted in relation to the strongest methods reported for the same parent study resource. In the BMMR2 challenge, the reference ACRIN 6698 benchmark achieved an AUC of 0.782, whereas the three highest-ranking submissions achieved AUC values of 0.803, 0.838, and 0.840 [11]. These approaches could incorporate multiparametric DCE and DWI information, alone or in combination, together with clinical characteristics. The AUROC of approximately 0.67 obtained in the present study is therefore lower than the strongest BMMR2 challenge results. However, direct comparison should remain cautious because the present analysis was deliberately restricted to one cropped-DCE branch and was designed primarily to establish a reproducible longitudinal preprocessing workflow and a controlled comparison between handcrafted radiomics and frozen deep image embeddings rather than to conduct an exhaustive multiparametric optimization. Accordingly, the present contribution lies in methodological reproducibility and representation-level comparison, not in achieving state-of-the-art predictive accuracy.

An AUROC of approximately 0.67 is insufficient for individual treatment modification, omission of surgery, or standalone classification of patients as responders or non-responders. The low sensitivity of several retained models at the prespecified operating threshold further precludes their current use as clinical screening or decision-support tools. The present results should therefore be interpreted as evidence of an exploratory imaging signal and a reproducible analytical workflow rather than as evidence of clinical readiness. At its present level of performance, the framework may support methodological development and hypothesis generation, but it has no direct role in individual clinical decision-making.

Several factors may explain why performance remained limited. The first is sample size. Although 183 patients is substantial for a manually curated longitudinal breast MRI study, the effective training subset comprised only 113 patients. This is a restrictive setting for stable high-dimensional modeling, particularly when feature construction includes multiple timepoints and longitudinal transformations. The class distribution further restricted the effective information available for learning the positive-response pattern, because the development subset contained only 35 pCR cases compared with 78 non-pCR cases. Although inverse-frequency class weighting was applied, this strategy could not fully compensate for the limited number and heterogeneity of pCR examples and may have contributed to the low sensitivity observed in several retained models. The second is cohort heterogeneity. Even within a controlled archive branch, lesion biology, treatment response patterns, imaging variability, and segmentation-related uncertainty may all contribute to noise. Third, the present study deliberately adopted a pragmatic first-stage configuration rather than an exhaustive or aggressively optimized one. Only one imaging branch was used, only four MIRP feature families were extracted in the handcrafted radiomics arm, the main longitudinal handcrafted descriptor was a direct T1–T0 delta formulation, and the deep-learning arm was limited to lesion-centered 2.5D inputs and frozen pretrained embeddings rather than multimodal or fully optimized end-to-end architectures. Alternative signal representations, especially those integrating more selective longitudinal structure or multimodal imaging inputs, may perform differently.

The current results also carry an important methodological implication. The failure of the raw merged handcrafted radiomic space to outperform the compact clinical baseline, followed by the partial recovery of signal after train-only feature selection, underscores the importance of disciplined analytical separation between data cleaning, feature engineering, feature selection, and model fitting. In small-to-moderate imaging cohorts, it is not enough to extract many descriptors or latent variables and rely on a flexible classifier to discover structure. The stability of the representation space itself becomes a central issue. For this reason, one of the key lessons of the present analysis is that longitudinal MRI modeling should be treated as a staged problem: first build the reproducible imaging cohort; then construct the quantitative descriptors or latent embeddings; then aggressively reduce non-informative variation using train-only logic; and only then interpret the biological content of the retained signal.

From a clinical perspective, the emergence of T1-only handcrafted radiomics as the strongest refined feature block is noteworthy. If confirmed in larger cohorts, this would suggest that the imaging phenotype observed after early treatment exposure may be more decision-relevant than a purely pretreatment representation. Such a possibility aligns conceptually with adaptive treatment monitoring, where the purpose is not only to characterize the lesion at baseline, but to identify whether therapy is already reshaping lesion burden, structure, and internal heterogeneity in a way that anticipates ultimate response. In this context, the present study offers a preliminary indication that the most informative handcrafted radiomic signal may reside in the early-treatment state itself rather than in crude handcrafted delta variables alone.

From a diagnostics perspective, these findings suggest that longitudinal breast MRI may contribute not only to response characterization, but also to early imaging-based risk stratification and non-invasive prognostic support during neoadjuvant therapy. If validated in larger cohorts, such imaging-derived signatures could help distinguish patients whose lesions already exhibit favorable early-treatment transformation from those whose residual phenotype remains more consistent with incomplete response, thereby supporting earlier and more informed treatment-monitoring decisions.

The comparison with the deep image-based branch adds an additional layer of interpretation to these findings. The formal paired comparison found no statistically significant difference in AUROC between the strongest handcrafted radiomics and frozen deep-embedding models (ΔAUROC = 0.002; 95% CI, −0.179 to 0.184; *p* = 0.988). The calibration analysis further showed that similar AUROC and AUPRC values did not translate into reliable absolute probability estimation. Both models demonstrated deviations from ideal calibration, and their predicted probabilities should therefore not be interpreted as clinically validated individual pCR-risk estimates. These findings reinforce the exploratory status of the models and the need for external recalibration and validation before any probability-based clinical interpretation. The observed differences should therefore be interpreted as representationally informative rather than as evidence of superiority of one modeling family over the other in the present exploratory cohort. Although the exploratory end-to-end paired T0/T1 deep learning models showed substantial overfitting and therefore could not be retained as the principal image-based strategy, the frozen pretrained embedding approach produced stable test-set performance that was broadly comparable to the strongest handcrafted radiomics benchmark. Importantly, the strongest signal was concentrated in different representation spaces across the two approaches. In the handcrafted radiomics branch, the best-performing refined model was based on the absolute early-treatment T1 phenotype, whereas in the frozen deep-embedding branch, the strongest AUROC was obtained from the DELTA-only latent representation and the strongest AUPRC from the AVG-only latent representation. This contrast suggests that handcrafted radiomic descriptors and pretrained deep image embeddings may not be redundant summaries of the same lesion information, but rather may emphasize different aspects of treatment response. In the present cohort, engineered radiomics appeared to capture the informative structure of the early-treatment lesion state itself, whereas frozen deep representations appeared to shift more of the predictive signal toward latent longitudinal change and integrated paired-image state. This divergence is methodologically important because it suggests that future hybrid models combining handcrafted and deep latent representations may be more informative than either representation family alone. This interpretation is also supported by recent breast MRI literature showing that deep learning, radiomics, and hybrid clinical-imaging models may capture partly distinct but complementary information during neoadjuvant treatment response assessment [23,26,27,28,29,30].

The study has several limitations. It was retrospective and entirely based on a public archive. The selected pipeline deliberately focused on one practical and coherent cropped-DCE branch and therefore did not incorporate the full multimodal richness of the source dataset, such as diffusion-weighted or ADC-derived branches. The analysis was also restricted to T0 and T1 to preserve an early-treatment-response prediction setting. Later T2 and T3 examinations may contain additional information related to cumulative treatment exposure and subsequent treatment evolution, but they were not evaluated in the present study and should be incorporated in future multitimepoint analyses. Requiring identifiable T0 and T1 image–segmentation pairs on the selected cropped-DCE VOLSER branch may also have introduced imaging-availability selection bias. Although no statistically significant differences were detected between the included and excluded patients in age, maximum lesion diameter, or pCR distribution, only eight patients were excluded. Consequently, the comparison had limited statistical power and cannot rule out clinically relevant systematic differences between the two groups. External validation was not performed; instead, the predefined BMMR2 split was preserved as an internal benchmark-style separation. In addition, multiple candidate configurations were compared using the same held-out benchmark subset. Although its labels did not influence preprocessing, feature ranking, hyperparameter optimization, model fitting, or checkpoint selection, repeated comparative evaluation may introduce model-comparison optimism. The reported headline performance estimates should therefore not be interpreted as unbiased confirmatory validation results. Although the predefined testing subset was fully held out from feature selection, hyperparameter optimization, model fitting, and checkpoint selection, it originated from the same ACRIN 6698/BMMR2 study resource and therefore does not constitute an external validation cohort. The reported performance may consequently remain specific to the cohort composition, acquisition conditions, segmentation procedures, and preprocessing characteristics of this dataset. Independent validation using patients acquired at other institutions and under different imaging protocols is required before the reproducibility, generalizability, or clinical utility of the models can be established. The sample size remained limited for high-dimensional supervised learning. The clinical feature block was also relatively compact, which constrains the strength of comparisons between imaging and non-imaging predictors. In addition, the present work evaluated only a first-stage longitudinal handcrafted delta construction strategy and a first-stage frozen embedding formulation and did not explore more advanced temporal modeling approaches. The exploratory end-to-end deep learning models also overfit substantially, and although the frozen embedding branch was more stable, it remained subject to the same small-sample and single-cohort limitations.

Future work should proceed in several directions. One priority is to evaluate whether the retained T1 handcrafted radiomic signal remains stable under alternative feature-selection schemes and simpler interpretable classifiers. Another is to test whether the DELTA and AVG deep embedding signals remain reproducible under alternative pretrained backbones and embedding extraction strategies. Multimodal integration, particularly with diffusion-related descriptors, ADC-derived features, or more targeted clinical covariates, may improve discrimination beyond the present results. More sophisticated longitudinal formulations may also be useful, for example those that model treatment-induced structural reorganization more explicitly than simple absolute or relative handcrafted feature differences or simple latent vector arithmetic. Finally, independent validation in external or expanded cohorts will be essential before any stronger translational claims can be made.

In summary, the present study shows that a complex public longitudinal breast MRI archive can be converted into a validated longitudinal modeling cohort for early treatment-response analysis, and that meaningful but modest predictive signal can be recovered from both handcrafted radiomic descriptors and frozen deep image embeddings within this framework. The strongest handcrafted result was concentrated in the early-treatment T1 radiomic phenotype, whereas the strongest deep representation results were concentrated in latent longitudinal change and integrated paired-image state. The resulting discrimination remains exploratory rather than clinically definitive, but the work nonetheless provides a reproducible longitudinal preprocessing and modeling pipeline for the BMMR2 archive and a data-driven indication that handcrafted and deep image-derived representations may capture complementary aspects of early treatment response.

## 5. Conclusions

In conclusion, the present study shows that a complex public longitudinal breast MRI archive can be transformed into a reproducible, analysis-ready cohort for early treatment-response modeling. Starting from the heterogeneous ACRIN 6698/BMMR2 resource, we established a validated 183-patient longitudinal dataset with matched T0 and T1 cropped DCE image–mask pairs, preserved train/test partitioning, binary pCR labeling, handcrafted radiomic feature extraction, and lesion-centered paired image tensors suitable for deep representation learning. This workflow itself is an important contribution because it converts a difficult public archive into a structured resource for comparative longitudinal imaging analysis.

Within this framework, the strongest handcrafted radiomics result was obtained from the early-treatment T1 feature block after train-only filtering, correlation pruning, and compact feature selection, indicating that the early-treatment lesion phenotype carried the clearest handcrafted imaging signal in the present cohort. In parallel, exploratory end-to-end paired T0/T1 deep learning models were technically feasible but showed marked overfitting and were therefore not retained as the principal image-based strategy. A frozen pretrained ResNet18 embedding approach produced more stable downstream results, with the strongest deep signal shifting toward DELTA and AVG latent representation blocks rather than the absolute T1 state alone.

Taken together, these findings suggest that handcrafted radiomics and frozen deep image embeddings may capture complementary aspects of early treatment response. The handcrafted branch appeared to emphasize the informative structure of the early-treatment lesion phenotype, whereas the deep embedding branch concentrated more signal in latent longitudinal change and integrated paired-image state. Although overall predictive discrimination remained modest, the present study provides a reproducible framework and preliminary evidence of complementary longitudinal imaging signals. The evaluated models are not suitable for individual clinical decision-making in their current form and require performance improvement and independent external validation before any translational application.

## Figures and Tables

**Table 1:** Cohort construction and pCR class distribution.

Cohort Stage	Overall, n	Training, n	Testing, n	Non-pCR, n	pCR, n
Full ancillary clinical spreadsheet	385	—	—	—	—
Initially eligible BMMR2 cohort	191	117	74	—	—
Complete T0/T1 cropped-DCE VOLSER pairs	183	113	70	128	55
Final training subset	113	113	—	78	35
Final independent testing subset	70	—	70	50	20

Note: Initial eligibility required predefined BMMR2 training or testing assignment, an available binary pCR label, and documented T0 and T1 examinations. Final inclusion additionally required complete T0/T1 image–segmentation pairs and successful phase-matched image–mask reconstruction.

**Table 2:** First-pass test-set performance of clinical, radiomic, and longitudinal feature representations for pCR prediction.

Feature Set	Model	n Features Used	AUROC	AUPRC	Accuracy	Precision	Sensitivity	Specificity	F1
Clinical only	Logistic regression	2	0.628	0.395	0.543	0.324	0.550	0.540	0.407
T0 + T1 radiomics	Random forest	166	0.623	0.351	0.671	0.333	0.150	0.880	0.207
T0 radiomics only	Random forest	83	0.613	0.360	0.657	0.333	0.200	0.840	0.250
T1 radiomics only	Gradient-boosted trees	83	0.608	0.373	0.671	0.364	0.200	0.860	0.258
T1 radiomics only	Random forest	83	0.605	0.393	0.629	0.200	0.100	0.840	0.133
T0 + T1 + delta radiomics	Random forest	331	0.580	0.350	0.686	0.375	0.150	0.900	0.214
Clinical + all imaging	Random forest	333	0.578	0.343	0.671	0.286	0.100	0.900	0.148
T0 + T1 radiomics	Gradient-boosted trees	166	0.570	0.349	0.686	0.417	0.250	0.860	0.313
T0 + T1 + delta radiomics	Gradient-boosted trees	331	0.561	0.368	0.671	0.333	0.150	0.880	0.207
Clinical + all imaging	Gradient-boosted trees	333	0.552	0.360	0.686	0.375	0.150	0.900	0.214
T0 radiomics only	Gradient-boosted trees	83	0.533	0.316	0.657	0.357	0.250	0.820	0.294
T1 radiomics only	Logistic regression	83	0.526	0.295	0.543	0.269	0.350	0.620	0.304
Delta radiomics only	Random forest	165	0.499	0.287	0.700	0.000	0.000	0.980	0.000
Clinical only	Gradient-boosted trees	2	0.487	0.285	0.586	0.200	0.150	0.760	0.171
Delta radiomics only	Gradient-boosted trees	165	0.482	0.292	0.671	0.000	0.000	0.940	0.000

**Table 3:** Test-set performance of the feature-selected radiomic models after train-only pruning and ranking.

Feature Set	Feature Budget	Model	AUROC	AUPRC	Accuracy	Sensitivity	Specificity	F1
T1 radiomics only	10	Random forest	0.670	0.405	0.643	0.200	0.820	0.242
T0 + T1 radiomics	20	Random forest	0.665	0.394	0.671	0.250	0.840	0.303
Clinical + T0 + T1	20	Random forest	0.655	0.400	0.686	0.250	0.860	0.313
Clinical only	2	Logistic regression	0.628	0.395	0.543	0.550	0.540	0.407

**Table 4:** Selected T1 radiomic features retained in the strongest imaging-based model.

Selected T1 Feature	Approximate Interpretation
T1_morph_volume	lesion size/tumor burden
T1_rlm_rlnu_3d_v_mrg_fbn_n32	run-length non-uniformity/texture heterogeneity
T1_morph_pca_least_axis	minor-axis extent/lesion thickness
T1_stat_cov	relative intensity dispersion
T1_stat_median	central lesion intensity level
T1_morph_comp_1	compactness-related morphology
T1_morph_pca_elongation	lesion elongation/anisotropy
T1_morph_vol_dens_conv_hull	lesion filling relative to convex hull
T1_morph_area_mesh	surface extent
T1_rlm_lrhge_3d_v_mrg_fbn_n32	long-run high-grey-level emphasis

**Table 5:** Quantitative summary of the exploratory end-to-end paired T0/T1 models. Both a compact custom CNN and a partially fine-tuned ResNet18 model were evaluated using an internal stratified validation subset and an independent 70-patient test subset. In both models, the optimal validation performance occurred during the first epoch, followed by divergence between training and validation behavior.

Model	Trainable Components	Best Epoch/Stopping Epoch	Best Validation AUROC	Test AUROC	Test AUPRC	Accuracy	Sensitivity	Specificity	F1
Compact custom paired CNN	Complete custom network	1/11	0.688	0.433	0.256	0.286	1.000	0.000	0.444
Partially fine-tuned paired ResNet18	Layer 4 and fusion head	1/9	0.643	0.504	0.353	0.714	0.000	1.000	0.000

Note: The independent test subset contained 50 non-pCR and 20 pCR cases. All operating-point metrics were calculated at the prespecified probability threshold of 0.5. The compact custom CNN predicted all 70 cases as pCR, whereas the partially fine-tuned ResNet18 predicted all 70 cases as non-pCR.

**Table 6:** Test-set performance of selected predictive models built on frozen pretrained ResNet18 image embeddings.

Feature Block	Feature Budget	Model	AUROC	AUPRC	Accuracy	Precision	Sensitivity	Specificity	F1
DELTA only	30	Random forest	0.672	0.408	0.657	0.300	0.150	0.860	0.200
AVG only	100	Logistic regression	0.666	0.472	0.686	0.458	0.550	0.740	0.500
DELTA only	50	Gradient-boosted trees	0.666	0.393	0.700	0.444	0.200	0.900	0.276
DELTA only	20	Logistic regression	0.641	0.431	0.600	0.375	0.600	0.600	0.462
T1 + DELTA	50	Gradient-boosted trees	0.636	0.397	0.686	0.333	0.100	0.920	0.154

**Table 7:** Comparison of the strongest handcrafted radiomics and frozen deep image-embedding models for pCR prediction.

Representation Family	Best Feature Block	Model	AUROC (95% CI)	AUPRC (95% CI)	Accuracy	Sensitivity	Specificity	F1	Main Representation Emphasis
Handcrafted radiomics	T1 only	Random forest	0.670 (0.513–0.811)	0.405 (0.319–0.607)	0.643	0.200	0.820	0.242	Early-treatment lesion phenotype
Frozen deep image embeddings (best AUROC)	DELTA only	Random forest	0.672 (0.529–0.812)	0.408 (0.314–0.610)	0.657	0.150	0.860	0.200	Longitudinal latent change
Frozen deep image embeddings (best AUPRC)	AVG only	Logistic regression	0.666 (0.529–0.806)	0.472 (0.343–0.671)	0.686	0.550	0.740	0.500	Integrated paired-image state

**Table 8:** Calibration metrics for the strongest handcrafted radiomics and frozen deep-embedding models on the predefined 70-patient held-out benchmark subset.

Model	Observed pCR Prevalence	Mean Predicted Probability	Brier Score	Calibration Intercept	Calibration Slope
T1-only radiomics RF, 10 features	0.286	0.318	0.211	−0.569	0.428
DELTA-only frozen embedding RF, 30 features	0.286	0.381	0.204	−0.294	1.340

Note: Lower Brier scores indicate better overall probabilistic accuracy. Ideal calibration corresponds approximately to an intercept of 0 and a slope of 1. Calibration intercepts and slopes were obtained through logistic recalibration for evaluation only; no post hoc recalibration was applied to the reported predictions. Because the benchmark subset contained only 70 patients, all calibration estimates are exploratory. RF, random forest; pCR, pathologic complete response.

## Data Availability

The imaging and clinical data analyzed in this study are publicly available from The Cancer Imaging Archive (TCIA) through the ACRIN 6698/I-SPY2 Breast DWI collection and the associated Breast Multiparametric MRI for Prediction of Neoadjuvant Chemotherapy Response 2 (BMMR2) Challenge resource, available at https://www.cancerimagingarchive.net/collection/acrin-6698/ (accessed on 17 June 2026) and https://wiki.cancerimagingarchive.net/pages/viewpage.action?pageId=89096426 (accessed on 17 June 2026). The processed image–mask pairs, extracted radiomic features, deep image representations, and other derived data supporting the findings of this study are available from the corresponding author upon reasonable request. No new primary patient data were prospectively collected for this study.

## Supplementary Materials

The following supporting information can be downloaded at: https://www.mdpi.com/article/10.3390/diagnostics16152349/s1, Table S1: Train-only probability-threshold sensitivity analysis for the highest-ranking targeted T1-only radiomics configuration; Table S2: Available clinical characteristics of patients included in and excluded from the final image-analysis cohort; Table S3: Observed and predicted pCR probabilities across five quantile-based calibration groups for the strongest handcrafted radiomics and frozen deep-embedding models.
