# Supplementary material for: Longitudinal Breast MRI for Early Treatment-Response Modeling: A Comparative Study of Handcrafted Radiomics and Frozen Deep Image Embeddings for pCR Prediction
Source: Diagnostics (Basel). 2026 Jul 27;16(15):2349. doi: 10.3390/diagnostics16152349 (PMC13464856; doi:10.3390/diagnostics16152349)
Supplement: Supplementary file 1 [file diagnostics-16-02349-s001.zip › diagnostics-4413532-supplementary.pdf]

Supplementary Materials

Table S1. Train-only probability-threshold sensitivity analysis for the highest-ranking targeted T1-only radiomics configuration.

Feature representation	Feature budget	Classifier	Threshold strategy	Threshold	Test AUROC	Test AUPRC	Accuracy	Sensitivity	Specificity	F1
T1-only radiomics	8	Random forest	Prespecified threshold	0.50	0.639	0.410	0.643	0.250	0.800	0.286
T1-only radiomics	8	Random forest	Train-only OOF Youden threshold	0.38	0.639	0.410	0.714	0.500	0.800	0.500

Note: The alternative threshold was selected exclusively from out-of-fold predictions generated within the 113-patient training subset using five-fold stratified cross-validation. Candidate thresholds from 0.20 to 0.80 were evaluated in increments of 0.02. The independent test labels were not used for threshold selection. AUROC and AUPRC are identical across the two rows because classification-threshold adjustment does not alter the ranking of predicted probabilities.

Table S2. Available clinical characteristics of patients included in and excluded from the final image-analysis cohort.

**Characteristic**	**Included cohort, n = 183**	**Excluded cohort, n = 8**
Training/testing subset, n	113/70	4/4
Age, median [IQR], years	48.00 [41.50–56.00]	44.50 [35.75–57.25]
Non-pCR/pCR, n	128/55	4/4
Maximum lesion diameter, median [IQR]	3.50 [2.80–5.05]	3.70 [3.23–5.05]
White, n	134	6
Black, n	18	0
Asian, n	13	0
Native Hawaiian or other Pacific Islander, n	1	0
Unknown race, n	14	1
Missing race, n	3	1
Single mass, n	70	3
Multiple masses, n	95	3
Single NME, n	7	2
Multiple NME, n	11	0
HR+/HER2−, n	82	2
HR−/HER2−, n	56	3
HR+/HER2+, n	32	1
HR−/HER2+, n	13	2
SBR grade I, n	5	0
SBR grade II, n	50	3
SBR grade III, n	127	5
Missing SBR grade, n	1	0

Note: Continuous variables are presented as median [interquartile range]. Age and maximum lesion diameter were compared using the Mann–Whitney U test, whereas pCR distribution was compared using Fisher's exact test. The remaining variables are presented descriptively because only eight patients were excluded. NME, non-mass enhancement; HR, hormone receptor; HER2, human epidermal growth factor receptor 2; SBR, Scarff–Bloom–Richardson.

Table S3. Observed and predicted pCR probabilities across five quantile-based calibration groups for the strongest handcrafted radiomics and frozen deep-embedding models.

**Model**	**Quantile group**	**Mean predicted probability**	**Observed pCR fraction**
T1-only radiomics RF, 10 features	1	0.102	0.071
T1-only radiomics RF, 10 features	2	0.205	0.214
T1-only radiomics RF, 10 features	3	0.265	0.286
T1-only radiomics RF, 10 features	4	0.359	0.500
T1-only radiomics RF, 10 features	5	0.658	0.357
DELTA-only frozen embedding RF, 30 features	1	0.275	0.071
DELTA-only frozen embedding RF, 30 features	2	0.332	0.286
DELTA-only frozen embedding RF, 30 features	3	0.361	0.214
DELTA-only frozen embedding RF, 30 features	4	0.416	0.500
DELTA-only frozen embedding RF, 30 features	5	0.520	0.357

Note: Patients were divided into five quantile-based groups according to the predicted pCR probability generated separately by each model. Each group contained 14 patients. The mean predicted probability represents the average model-estimated probability within each group, whereas the observed pCR fraction represents the proportion of patients who achieved pCR. These numerical values correspond to the points displayed in Figure 6 of the main manuscript. RF, random forest; pCR, pathologic complete response.
